# Gut Microbiota-Derived SCFAs Modulated by Different Structures of Marine Algal Oligosaccharides Alleviate Colitis Through PPARγ Signaling Activation

**DOI:** 10.3390/nu18172750

**Published:** 2026-08-22

**Authors:** Jiang Li, Jiaojiao Tan, Junjie Liu, Minghui Wang, Xiaoqian Gu, Zhiyan Wang, Jican Han, Chaofan Pan, Ting Fu, Shirong Xue, Long Chen, Xinning Pan

**Affiliations:** 1 Marine Bioresource and Environment Research Center, First Institute of Oceanography, Ministry of Natural Resources, Qingdao 266061, China; guxiaoqian5843@163.com (X.G.); wangzhiyan@fio.org.cn (Z.W.); chenlong@fio.org.cn (L.C.); pxn7777777@163.com (X.P.); 2 School of Medicine, Qingdao Huanghai University, Qingdao 266427, China; qdrdtanjiaojiao@163.com (J.T.); liujj11@qdhhc.edu.cn (J.L.); wangmh02@qdhhc.edu.cn (M.W.); 17562716640@163.com (J.H.); kilig_1020@163.com (C.P.); 15006736525@163.com (T.F.); 17860778390@163.com (S.X.)

**Keywords:** marine algal oligosaccharides (MAOs), structure-function relationship, anti-inflammatory, colitis, SCFA, PPARγ

## Abstract

**Background:** Marine algal oligosaccharide (MAO) molecular structural characteristics are closely related to their bioactivity. However, an understanding of the specific relationships between MAO molecular structure (including polymerization degree [DP] and sulfate groups) and bioactivity is lacking. **Methods:** In this work, laboratory-prepared DP4 and DP8 MAOs, including agar- (AOs) and κ-, ι-, and λ-carrageenan-oligosaccharides (COs), were examined for their anti-inflammatory activity in RAW264.7 macrophages and therapeutic effect on colitis in mice; additionally, the potential mechanisms involved were explored. **Results:** The results showed that CO (ι- and λ-COs) anti-inflammatory activity was higher than that of AOs and was positively correlated with sulfate group content. MAOs with different structures all alleviated colitis symptoms; those with higher sulfate group content and DP showed excellent therapeutic efficacy. MAOs modulated gut microbiota and promoted the enrichment of microbiota-derived short-chain fatty acid production (butyrate), which potentially induced PPARγ signaling and contributed to the impacts of MAOs on mucosal homeostasis restoration. **Conclusions:** Therefore, MAOs with higher sulfate content and DP showed improved therapeutic efficacy compared with those with lower contents, which involved higher SCFA production.

## 1. Introduction

Marine algal oligosaccharides (MAOs) are prepared from algal polysaccharides, such as agar, carrageenan, alginate, and fucoidan, through physical, chemical, and enzymatic hydrolysis. MAOs possess various biological activities, including immuno-modulatory, anti-inflammatory, anti-oxidant, anti-tumor [1], and prebiotic effects [2], and have garnered increasing attention in functional foods, agriculture, and biomedicine.

Red seaweed, which is rich in agar and carrageenan, is one of the most abundant sources for oligosaccharide preparation, mainly including agar- (AOs) and carrageenan-oligosaccharides (COs). Generally, MAO bioactivities are closely related to their molecular structure, including polymerization degree (DP), position of glycosidic linkages, and sulfate group content [3,4]. AOs have a repeating disaccharide unit of 3-linked β-D-galactopyranose and 4-linked α-galactopyranose residues; κ-COs are slightly different, being composed of repeating disaccharide units of 3-linked 4-sulfate-β-D-galactopyranose and 4-linked 3,6-anhydro-L-galactose. ι- and λ-COs have a similar repeating disaccharide to κ-COs, but with two and three sulfate groups, respectively [1].

The relationship between sulfate group content and MAO biological activity remains unclear, though they are closely related. Yao et al. prepared κ-COs and their desulfurized derivatives using enzymatic hydrolysis that inhibited tumor necrosis factor-α release in microglia activated by lipopolysaccharide (LPS), with superior anti-inflammatory activity to their desulfurized derivatives [5]. Guo et al. reported that the anti-inflammatory activity of κ-COs increased with increasing DP, while for the same DP, the activity of oligosaccharides with more sulfate groups was higher [6]. These findings indicate that DP also plays a role in MAO biological activity. In examining the anti-inflammatory activities of AOs (NA2, NA4, NA6, NA8, and NA10), NA4 showed the most suppressed NO production by inhibiting the expression and secretion of iNOS and pro-inflammatory cytokines (interleukin [IL]-1β and IL-6) [7]. However, the specific relationship between MAO molecular structure and anti-inflammatory activity requires further study.

MAOs, similar to some NDOs (also called functional oligosaccharides), can act as prebiotics, offering a significant benefit to host health by stimulating beneficial bacterial growth and modulating colonic microbiota composition [8]. In addition, most MAOs are resistant to enzymatic degradation in the stomach and small intestine; they are instead fermented by gut bacteria and converted into short-chain fatty acids (SCFAs) and lactic acid [9,10]. SCFAs act on peripheral tissues and maintain intestinal barrier function by improving mucosal integrity, preventing symptom relapse in inflammatory bowel diseases (IBDs) [11]. Therapeutic modification of the gut microbiota by MAOs, in addition to their excellent anti-inflammation activity, appears to be a more promising strategy than current therapies for colitis. MAOs have potential health-promoting effects on intestinal functions; however, data on the anti-inflammatory activity of ι- and λ-COs are lacking, and the underlying mechanism of MAOs in colitis prevention and treatment is unclear. There is a need for evaluating colitis prevention and treatment with MAOs with different structural characteristics.

A series of MAOs, including AOs and κ-, ι-, and λ-COs with different DPs (DP4 and DP8), were prepared in this study using our self-developed macroalgae polysaccharide-degrading enzyme to investigate their anti-inflammatory activity and therapeutic effect on DSS-induced colitis in mice. Additionally, the underlying mechanisms involved were revealed.

## 2. Materials and Methods

### 2.1. MAO Preparation

MAOs, including AOs and κ-, ι-, and λ-COs with different DPs (DP4 and DP8), were prepared in our laboratory as described previously [12,13]. In brief, purified recombinant enzymes, including agarase (Aga1365 [12]), κ-carrageenase (Car3231 [13], ι-carrageenase [13]), and λ-carrageenase (Car3193 [13]), were added to 0.1% (*w*/*v*) corresponding substrate solutions of agarose (Shanghai Baygene Biotechnology Co., Ltd., Shanghai, China), κ-carrageenan, ι-carrageen, and λ-carrageen (Tokyo Chemical Industry Co., Ltd., Tokyo, Japan) under the optimal reaction temperatures and pHs. The reaction products were centrifuged at 9700× *g* for 20 min and the supernatant was collected for purification.

Reaction product compositions were analyzed with thin-layer chromatography (TLC). TLC plates were developed with an n-butanol–acetic acid–water solution (1:2:1, *v*/*v*), and oligosaccharide spots were visualized by spraying with 10% (*v*/*v*) H_2_SO_4_ in ethanol and heating at 80 °C for 15 min. Lyophilized products were further analyzed by HPLC. Samples were dissolved in 5 mg/mL Na_2_SO_4_, filtered (0.45 µm membrane), and analyzed with a TSKgel G2500PWxL column (7.8 mm I.D., 30 cm length; Tosoh Bioscience LLC, King of Prussia, PA, USA). The mobile phase was 5 mg/mL NaSO_4_, supplied at 500 μL/min.

### 2.2. MAO Purification

Products were collected after different reaction times and mixed with a 3× volume of absolute ethanol at 4 °C for 24 h. The supernatant was centrifuged at 9700× *g* for 15 min and concentrated using vacuum-rotary evaporation at 55 °C. The concentrates were filtered through a 0.22-μm membrane and purified using gel-filtration chromatography on a Bio-Gel P-4 column (100 × 2 cm). The column was eluted with distilled water using a pump with a flow rate of 0.2 mL/min, and 4 mL fractions were collected. The eluted fractions were pooled together using the phenol–sulfuric acid method and lyophilized for further analysis.

### 2.3. Structural Analysis

Purified oligosaccharide samples were analyzed using a time-of-flight mass spectrometer (Agilent 6560, Santa Clara, CA, USA) equipped with an electrospray ionization source as described by Sun et al. [14]. In brief, lyophilized samples were dissolved in ultrapure water and filtered through a 0.22 μm membrane. Samples of 10 μL (1 mg/mL) were injected through a 2 μL Rheodyne loop, with 50% aqueous methanol used as the mobile phase with a flow rate of 200 μL/min. The electrospray ion spray voltage was set at 4 kV, with a sheath gas (nitrogen) flow rate of 30 arbitrary units, an auxiliary gas (nitrogen) flow rate of 5 arbitrary units, a tube lens voltage of −250 V, a capillary temperature of 350 °C, and a capillary voltage of −48 V.

### 2.4. Anti-Inflammatory Assay with LPS-Stimulated Macrophages

#### 2.4.1. Materials and Instruments

RAW264.7 macrophages (CL-0190) were purchased from Punosai Life Technology Co., Ltd. (Wuhan, China). Dulbecco’s modified Eagle medium (DMEM), fetal bovine serum, phosphate-buffered saline (PBS, pH7.0), and penicillin-streptomycin solution were purchased from Gibco (Grand Island, NY, USA). LPS, dexamethasone (DXM), and dimethyl sulfoxide were purchased from Sigma-Aldrich (St. Louis, MO, USA). The cell counting kit-8 and ECL luminescence detection kit were purchased from Beyotime (Nantong, Jiangsu, China). The enzyme-linked immunosorbent assay kit was purchased from Shanghai Enzyme-linked Biotechnology Co., Ltd. (Shanghai, China). All other reagents were analytical grade.

The main instruments included a fluorescence microscope (Olympus, Hachioji, Japan), a CO_2_ cell incubator, a high-speed low-temperature centrifuge, and a −80 °C freezer (Thermo Fisher Scientific, Waltham, MA, USA), a microplate reader (BioTeK, Winooski, VT, USA), an ice machine (Sanyo, Osaka, Japan), and an electrophoresis apparatus and gel imaging system (Bio-Rad, Hercules, CA, USA).

#### 2.4.2. Cell Culture and Cytokine Assay

RAW264.7 macrophages were cultured in DMEM with 10% (*v*/*v*) fetal bovine serum and 1% (*v*/*v*) antibiotics (100 U/mL penicillin and 100 U/mL streptomycin) in a 5% CO_2_ humidified incubator at 37 °C. Cells in the logarithmic growth phase were inoculated into a 96-well plate at a cell density of about 4 × 10^4^ cells/mL and treated with different MAOs (50, 200, and 400 μg/mL) and LPS (1 μg/mL) for 24 h. Cells treated with DXM (50 μg/mL) and LPS (1 μg/mL) were used as a positive drug control group; those treated with LPS (1 μg/mL) served as an inflammation model group, and DMEM only was used as a blank control. After 24 h of cultivation, 50 μL of cell supernatant was used to determine TGF-β1, IL-4, IL-6, and IL-10 concentrations using a commercial enzyme-linked immunosorbent assay kit. All experiments were conducted with three parallel treatments.

### 2.5. Therapeutic Effect on DSS-Induced Colitis Mice

#### 2.5.1. Reagents and Animals

A total of 88 specific pathogen-free C57BL/6 mice (male) were purchased from Hangzhou Ziyuan Experimental Animal Technology Co., Ltd. (SCK (ZHE) 2019-0004, Hangzhou, China). Mice were housed individually in Makrolon cages and maintained in an air-conditioned atmosphere with a 12 h light–dark cycle for 3 days with unrestricted access to regular food and water. All animal operations strictly followed the principles of random grouping, blind testing and animal welfare ethics. The entire animal experimental protocol was reviewed and approved by the Biomedical Ethics Committee of Qingdao Huanghai University (Approval Number: HHSYLL-2024-0601).

Mouse diet (Medison, MD12033) was purchased from Suzhou Medison BioMedicine Co., Ltd., Suzhou, China. The components of the mouse diet consist of casein (25.84%), maltodextrin (16.15%), sucrose (8.89%), cellulose (6.46%), soybean oil (3.23%), lard (31.66%), and mineral-vitamin premix (7.77%). DSS (40 kDa) was purchased from Aladdin Biochemical Technology Co., Ltd. (Shanghai, China). ACTIN rabbit (GB15003), GAPDH rabbit (GB15004), claudin-1 rabbit (GB112543), occludin rabbit (GB111401), ZO-1 rabbit (GB111402), and PPARγ rabbit (GB11164) polyclonal antibodies, as well as horseradish peroxidase goat anti-rabbit (GB23303), were obtained from Wuhan Servicebio Technology (Wuhan, China). 4′,6-Diamidino-2-phenylindole (C1005) was purchased from Beyotime (Shanghai, China).

#### 2.5.2. Experimental Design

Male C57BL/6 mice (8 weeks old, approximately 20 g) were randomly assigned to 11 groups (*n* = 8), including a control, DSS, positive control, and eight MAO treatment groups. After acclimatization, animals were subjected to stratified block randomization based on body weight. All animals were ranked by body weight and divided into multiple blocks. Within each block, animals were randomly assigned to different groups using random number generation to achieve balanced baseline body weight among groups. The MAO treatment groups were administered doses of 50 mg/kg·day for 3 weeks, based on preliminary experiments. At the third week, mice were administered 3% DSS (*w*/*v*) in drinking water for 7 consecutive days to induce colitis. The DSS model group was given phosphate-buffered saline solution for 3 weeks. The positive control group was given sulfasalazine (100 mg/kg·day) in drinking water for 3 weeks. The blank control group was orally administered PBS solution for 3 weeks. Body weight gains were recorded, and feces were observed. The disease activity index [15] was determined using the criteria described in Table 1. All blood samples were collected under deep anesthesia prior to euthanasia after being denied food and drink for 24 h, and colon tissues were collected for further examination. All 88 animals survived to the termination of the experiment without mortality or exclusion.

#### 2.5.3. Colonic Histological Staining

The colonic tissues were immediately washed with PBS, preserved with 4% paraformaldehyde for 48 h, and embedded in paraffin. The embedded samples were cut into 5–6 μm thick slices, dewaxed with xylene, and stained with hematoxylin and eosin to observe the colonic tissue morphology [16,17]. The colonic histological score was determined using the criteria described in Table 2.

#### 2.5.4. Immunohistochemical Assay

Colonic tissue was assessed using indirect immunofluorescence to observe tight junction proteins [18]. Briefly, slices were blocked with 5% bovine serum albumin and incubated with specific primary antibodies and the corresponding fluorescent secondary antibodies (Cell Signaling Technology, Danvers, MA, USA) overnight at 4 °C. After 4′,6-diamidino-2-phenylindole staining for 10 min in the dark, images were captured using a fluorescence microscope (Nikon Eclipse C1).

#### 2.5.5. RNA Sequencing

Total RNA was extracted from colon tissues with TRIZOL reagent (Invitrogen, Carlsbad, CA, USA). RNA sequencing libraries were constructed using the TruSeq™ RNA Sample Prep kit (Illumina, San Diego, CA, USA) according to the manufacturer’s instructions. Sequencing was performed by Beijing Novogene Biotechnology Co., Ltd. (Beijing, China) on an Illumina HiSeq2000 instrument. Raw reads were filtered to remove low-quality sequences and adapter contamination. Quality metrics and genome alignment results are summarized in Tables S1 and S2. Gene quantification was carried out using featureCounts within the Subread package. Reads with mapping quality <10, unpaired mapped reads and multi-mapped reads were filtered prior to subsequent analysis. GO functional enrichment and KEGG pathway enrichment analyses of differentially expressed genes were implemented using the clusterProfiler package. Differentially expressed genes (DEGs) were screened using the DESeq2 package. Raw read counts were normalized, followed by P-value calculation based on the negative binomial distribution. The Benjamini–Hochberg (BH) procedure was adopted for multiple hypothesis testing correction to obtain FDR values. DEGs were filtered according to the criteria |log_2_(FoldChange)| ≥ 1 and padj ≤ 0.05. The RNA-seq data have been deposited in the NCBI database (GenBank accession number: PRJNA1513481).

#### 2.5.6. Intestinal Microflora and SCFA Analysis

Microbial DNA was extracted from fecal samples using a FastDNA Spin Kit for Soil (MP Biomedicals, Santa Ana, CA, USA), according to the manufacturer’s instructions. DNA was quantified using a NanoDrop 2000 spectrophotometer (Thermo Fisher Scientific, Waltham, MA, USA). The V3–V4 hypervariable regions of the microbial 16S rRNA gene were amplified by PCR using primers 341F (5′-CCTAYGGGRBGCASCAG-3′) and 806R (5′-GGACTACNNGGGTATCTAAT-3′). The amplicon library was purified and sequenced on an Illumina MiSeq sequencing-by-synthesis platform (Novogene Bioinformatics Technology Co., Ltd., Beijing, China). Raw data were combined and filtered into clean reads using QIIME2. Operational taxonomic units (OTUs) were clustered at 97% similarity using the uclust OTU selection method, and the most abundant reads from each OTU were aligned using the Py-NAST algorithm. Data quality control results are summarized in Table S3.

Alpha-diversity, including Chao1, Shannon, and richness, was evaluated using QIIME2 [19]. Gut microbial community differences were analyzed with non-metric multidimensional scaling based on the Bray–Curtis distance. Linear discriminant analysis coupled with effect size measurements analysis was performed using the Kruskal–Wallis rank sum test to detect microbial taxa with significantly different abundances among groups; linear discriminant analysis was used to estimate the effect size of each taxon [20].

Gut microbiota SCFAs were determined using ultra-high performance liquid chromatography coupled to tandem mass spectrometry (Vanquish™ Flex UHPLC-TSQ Altis™, Thermo Scientific Corp., Dreieich, Germany). Fecal metabolite extraction and mass spectrometry parameters followed the methods of Han et al. [21]. All SCFA standards were obtained from ZZ Standards Co., LTD. (Shanghai, China).

#### 2.5.7. Western Blot Assays

Colonic tissue samples were homogenized in RIPA lysis buffer (1% PMSF), and the protein concentration was determined using a BCA kit (BL521A, Biosharp, Hefei, Anhui, China) according to the kit instructions. After centrifugation, the proteins were separated using sodium dodecyl sulfate-polyacrylamide gel electrophoresis (SDS-PAGE, 15% gels) and electrotransferred onto polyvinylidene fluoride membranes. The membranes were blocked for 1 h and incubated overnight at 4 °C with primary antibodies against ZO-1, occludin, claudin-1, and PPARγ at a dilution of 1:1000. Subsequently, secondary antibodies (1:3000) were added for 2 h. Protein bands were visualized by BeyoECL (Bio-Rad, Hercules, CA, USA); expression levels were quantified using ImageJ version 1.52 (NIH, Bethesda, MD, USA).

#### 2.5.8. Modeling of Butyrate Binding to PPARγ

For modeling the structure of the complex between PPARγ and SCFAs (acetic acid, propionic acid, and butyrate), Autodock Vina 1.2.2 docking software (The Scripps Research Institute, Hercules, CA, USA) was used to assess PPARγ-SCFA interactions. The X-ray crystal structure of PPARγ and PPARα with bound rosiglitazone (PDB 1FM6) was used to identify the likely SCFA binding site, where PPARα and rosiglitazone protein residues were deleted [22]. The molecular docking results were then visualized and analyzed using PyMOL 2.4 software (Schrödinger, New York, NY, USA).

#### 2.5.9. Statistical Analysis

All quantitative data were represented as mean ± standard error of the mean, and statistical analyses were performed using SPSS 27.0 software, and statistical graphs were generated by GraphPad Prism 9. The normality of data was assessed using the Shapiro–Wilk test, and homogeneity of variance was evaluated by the Levene test. For data that met the assumptions of normality and homogeneity of variance, one-way ANOVA was performed, followed by Tukey’s post hoc multiple-comparison test. Otherwise, the Kruskal–Wallis test was adopted, and Dunn’s test was used for pairwise comparisons among groups. A *p*-value < 0.05 was considered statistically significant, while a *p*-value < 0.01 was considered highly statistically significant.

## 3. Results

### 3.1. MAO Structural Analysis

**Figure 1:**
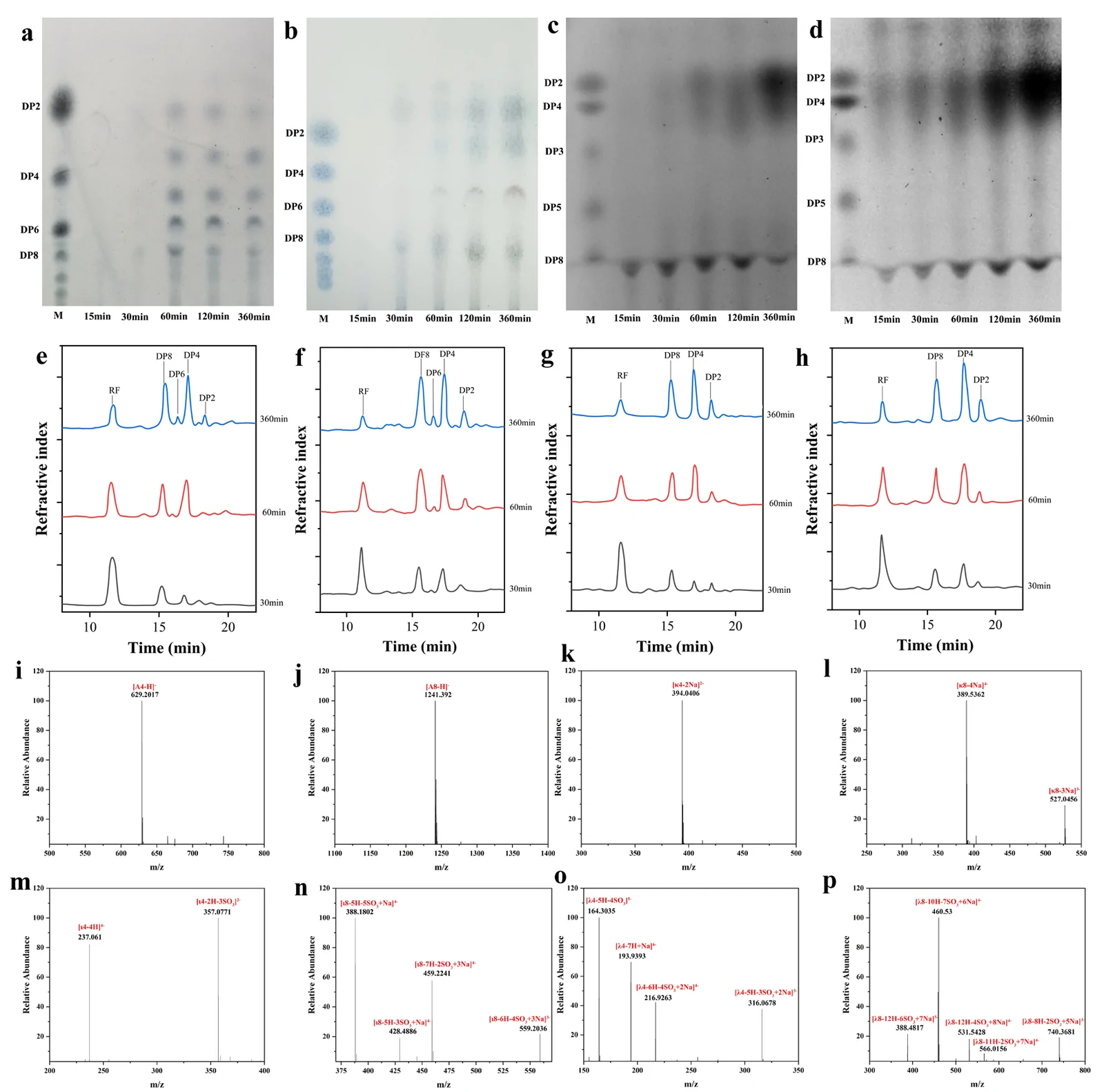
TLC spectra of MAOs degraded by different macroalgae polysaccharide-degrading enzymes (MAPD). (**a**) Agarase Aga1365; (**b**) κ-carrageenanase Car3231; (**c**) ι-carrageenase Car3206; (**d**) λ-carrageenase Car3193. HPLC spectra of MAOs degraded by different MAPDs; (**e**) agarase Aga1365; (**f**) κ-carrageenanase Car3231; (**g**) ι-carrageenase Car3206; (**h**) λ-carrageenase Car3193. TOF-MS spectra of different MAOs; (**i**) neoagarotetraose (A-4); (**j**) neoagaroctasaccharide (A-8); (**k**) κ-carrotetraose (κ-4); (**l**) κ-carroctasaccharide (κ-8); (**m**) ι-carrotetraose (ι-4); (**n**) ι-carroctasaccharide (ι-8); (**o**) λ-carrotetraose (λ-4); (**p**) λ-carroctasaccharide (λ-8).

TLC showed the main products of agarase Aga1365, κ-carrageenase Car3231, ι-carrageenase Car3206, and λ-carrageenase Car3193 against corresponding substrates, including neoagaroctasaccharide, neoagarohexaose, neoagarotetraose, and neoagarobiose (Figure 1a); κ-carroctasaccharide, κ-carrohexaose, κ-carrotetraose, and κ-carrobiose (Figure 1b); ι-carroctasaccharide, ι-carrotetraose, and ι-carrobiose (Figure 1c); and λ-carroctasaccharide, λ-carrotetraose, and λ-carrobiose (Figure 1d), respectively. The above results were further confirmed by HPLC (Figure 1e–h).

To obtain MAOs with different DPs, different elution peaks of corresponding enzymatic hydrolysis products were collected and identified using time-of-flight-mass spectrometry. Typical peaks were obtained for different oligosaccharide samples. A peak at *m*/*z* 629.2017 was assigned to [A4-H]^−^, representative of neoagarotetraose, indicating that NA4 was the dominant component of this sample (Figure 1i); a peak at *m*/*z* 1241.392 was assigned to [A8-H]^−^, representing neoagaroctasaccharide (Figure 1j), and a peak at *m*/*z* 394.0406 was assigned to [κ4-2Na]^2−^, representing κ-carrotetraose (Figure 1k). Peaks at *m*/*z* 389.5362 and 527.0456 were assigned to [κ8-4Na]^−^ and [κ8-3Na]^−^, respectively, representing κ-carroctasaccharide (Figure 1l). Peaks at *m*/*z* 237.061 and 357.0771 were assigned to [ι4-4H]^4−^ and [ι4-2H-3SO_3_]^2−^, respectively, representing ι-carrotetraose (Figure 1m). Peaks at *m*/*z* 388.1802, 428.4886, 459.2241, and 559.2036 were assigned to [ι8-5H-5SO_3_ + Na]^4−^, [ι8-5H-3SO_3_ + Na]^4−^, [ι8-7H-2SO_3_ + 3Na]^4−^, and [ι8-6H-4SO_3_ + 3Na]^3−^, respectively, representing ι-carroctasaccharide (Figure 1n). Peaks at *m*/*z* 164.3035, 193.9393, 216.9263, and 316.0678 were assigned to [λ4-5H-4SO_3_]^5−^, [λ4-7H + Na]^6−^, [λ4-6H-4SO_3_ + 2Na]^4−^, and [λ4-5H-3SO_3_ + 2Na]^3−^, respectively, representing λ-carrotetraose (Figure 1o). Peaks at *m*/*z* 388.4817, 460.53, 531.5428, 566.0156, and 740.3681 were assigned to [λ8-12H-6SO_3_ + 7Na]^5−^, [λ8-10H-7SO_3_ + 6Na]^4−^, [λ8-12H-4SO_3_ + 8Na]^4−^, [λ8-11H-2SO_3_ + 7Na]^4−^, and [λ8-8H-2SO_3_ + 5Na]^3−^, respectively, representing λ-carroctasaccharide. These data showed that different AOs and COs, including neoagarotetraose (A-4), neoagaroctasaccharide (A-8), κ-carrotetraose (κ-4), κ-carroctasaccharide (κ-8), ι-carrotetraose (ι-4), ι-carroctasaccharide (ι-8), λ-carrotetraose (λ-4), and λ-carroctasaccharide (λ-8), were successfully prepared.

### 3.2. Effects of MAOs on LPS-Induced RAW264.7 Cells

**Figure 2:**
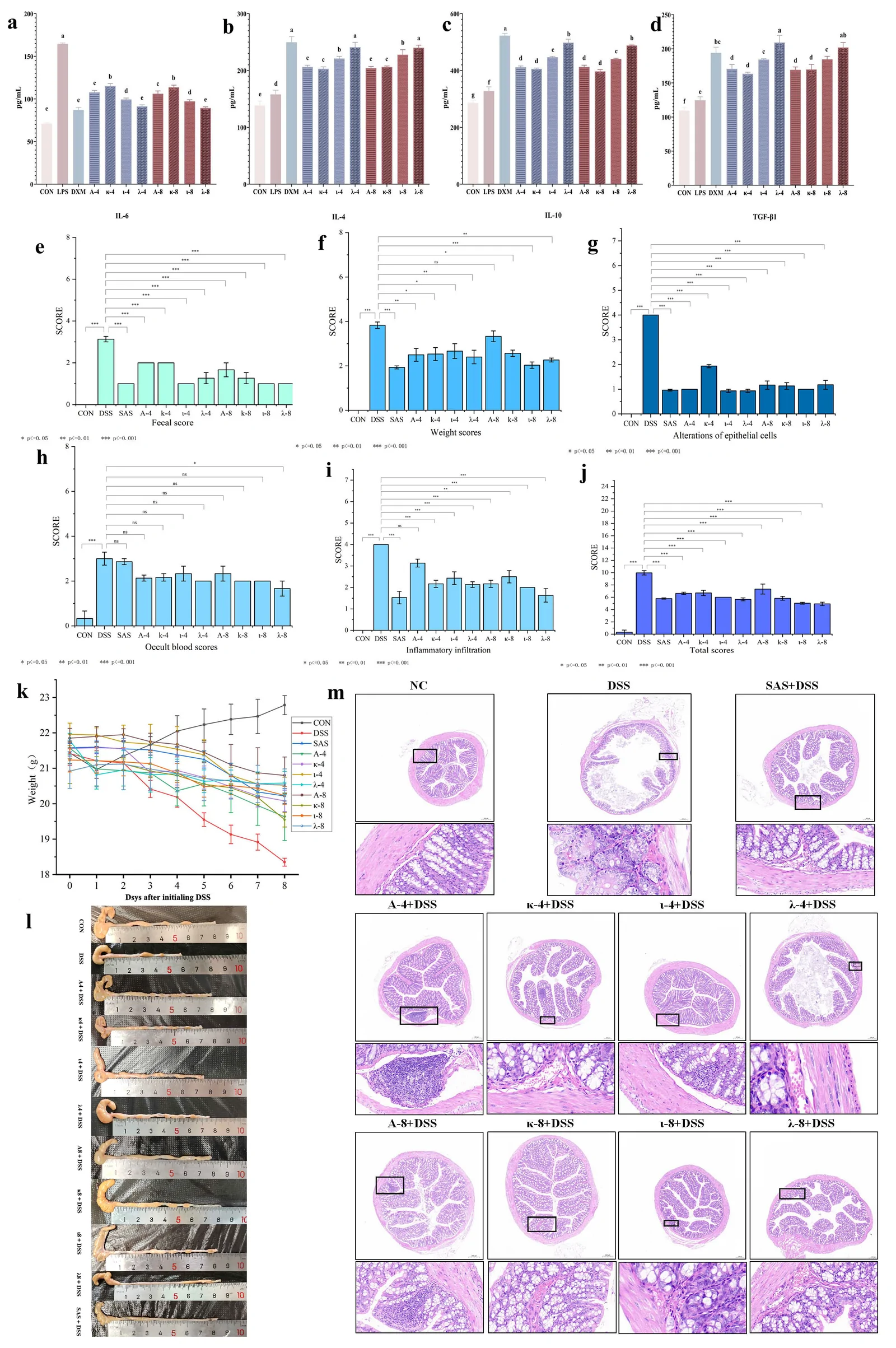
The cytokine concentration in lipopolysaccharide-included RAW264.7 cells (*n* = 3). (**a**) Interleukin (IL)-6; (**b**) IL-4; (**c**) IL-10; and (**d**) transforming growth factor (TGF)-β1. Different lowercase letters in (**a**–**d**) represent significant differences between respective treatments by ANOVA (*p* < 0.05). The DAI indicators (*n* = 8). (**e**) Fecal score; (**f**) weight score; (**g**) alterations of epithelial cells score; (**h**) occult blood scores; (**i**) inflammatory infiltration score; (**j**) total score. (**k**) The body weight; (**l**) the colon lengths; (**m**) H&E staining of colon (scale: 200 μm).

All oligosaccharide samples inhibited the expression of the inflammatory factor IL-6 (*p* < 0.05 or 0.01); additionally, anti-inflammatory factors (IL-4, IL-10, and TGF-β1) were significantly enhanced (*p* < 0.05 or 0.01) compared with LPS control groups, but were slightly lower than the positive control group (DXM) (Figure 2a–d).

Sulfate groups had a more significant effect on cytokine expression, with CO anti-inflammatory activity generally being higher than that of AOs. The anti-inflammatory activity of high-sulfate oligosaccharides (ι- and λ-COs) was significantly higher (*p* < 0.01, *p* < 0.01) than that of AOs without sulfate groups. Anti-inflammatory activity was significantly positively correlated with CO sulfate group content. IL-6 level was significantly decreased (*p* < 0.01) by pretreatment with different DP κ-, ι-, and λ-COs at 200 μg/mL, decreasing more with increasing sulfate group content (Figure 2a). IL-4, IL-10, and TGF-β1 levels were significantly (*p* < 0.01) increased by pretreatment with different DP κ-, ι-, and λ-COs at similar concentrations, increasing with increasing sulfate group content (Figure 2b–d). However, there were no statistical anti-inflammatory activity differences between different DPs of the same type of oligosaccharide.

### 3.3. MAO Treatment Alleviated DAI Indicators

MAOs significantly alleviated symptoms in DSS-induced mice colitis after 3 weeks of administration, including weight loss, fecal morphology, and fecal occult; induced colitis mice showed typical colitis symptoms (Figure 2e–j). All MAO-treated groups had significantly less weight loss caused by DSS-induced colitis (average: <7%), while that of the DSS group was about 15%. The weight loss range of groups treated with low-DP MAOs (λ-4, ι-4, κ-4, and A-4) was between 5.49% and 8.94%, while that of mice treated with high-DP MAOs (λ-8, ι-8, κ-8, and A-8) was between 1.86% and 4.76%. Weight loss alleviation for high-sulfate MAOs (ι- and λ-COs) was greater than that of κ-COs and AOs, and that of high-DP MAOs (DP8) was greater than that of low-DP MAOs (DP4) (Figure 2k).

### 3.4. MAO Treatment Ameliorated Colon Length Changes

Changes in colon length are important indicators for evaluating colitis. After 1 week of DSS induction, the colon length of the DSS group was 6.57 ± 0.66 cm, significantly shorter (*p* < 0.01) than that of the normal group (average 7.88 ± 0.34 cm); the colon length of the oligosaccharide-treated group was longer than that of the DSS group (*p* < 0.05) (Figure 2l). Colon lengths of the low-DP MAO groups (λ-4, ι-4, κ-4, and A-4) were 7.70 ± 0.57, 7.23 ± 0.54, 7.40 ± 0.53, and 7.75 ± 0.68 cm, respectively, while those of the high-DP MAO groups (λ-8, ι-8, κ-8, and A-8) were 8.08 ± 0.69, 8.24 ± 0.72, 7.93 ± 0.39, and 7.70 ± 0.73 cm. For COs, colon lengths of high-sulfate MAO groups (ι- and λ-COs) were longer than those of κ-CO groups. Alleviation of colitis-induced colon length decrease by high-DP MAOs was better than that of low-DP MAOs for the same oligosaccharide type.

### 3.5. MAO Treatment Repaired Colitis Mucosal Structure

Colonic epithelial cells of the blank control group had tight junctions, gland structure was normal, and there was no inflammatory cell infiltration (Figure 2m). Samples from the DSS group showed severe colonic epithelial cell depletion (*p* < 0.01), glandular structure disorder, submucosal edema, and inflammatory cell infiltration (*p* < 0.01).

Colon epithelial cells of MAO-treated mice were arranged tightly, and the glandular structure was normal; only the κ-4 treatment group showed significant colon epithelial and goblet cell depletion, submucosal edema, and diffuse glandular structures (Figure 2m). Mice treated with high-sulfate MAOs (ι- and λ-COs) had only slight lymphocyte infiltration in the mucosal lamina propria; however, groups treated with κ-COs and AOs had significant neutrophil infiltration, especially in the mucosal muscle layer. MAO administration significantly alleviated the damage to colon epithelial tissue caused by DSS, with high-sulfate MAOs reducing inflammatory cell infiltration more than κ-COs and AOs.

### 3.6. MAO Treatment Alleviated Inflammatory Responses in Colitis Mice

Colitic mice treated with MAOs had significantly reduced IL-6 expression but increased IL-4, IL-10, and TGF-β1 expression in colonic tissue (Figure 6e). The correlation between the molecular structure (including DP and sulfate group number) and MAO anti-inflammatory activity in serum was similar to that of the RAW264.7 macrophage treatment. MAO pretreatment in mice with DSS-induced colitis significantly altered the immune response to alleviate colonic inflammation.

### 3.7. MAOs Protected Against DSS-Induced Intestinal Barrier Dysfunction

**Figure 3:**
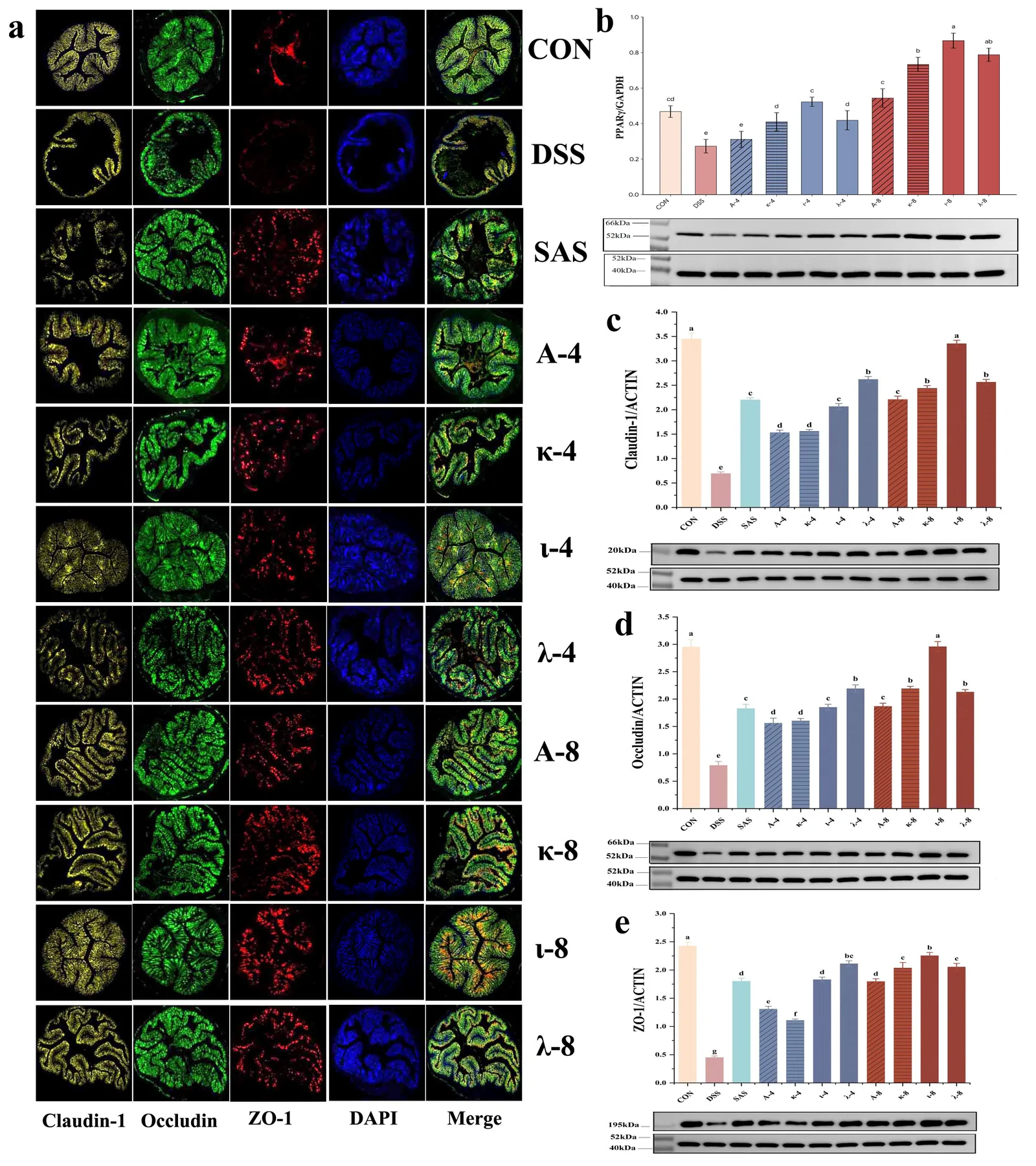
Immunofluorescence analysis of the expression of tight junction proteins in colon tissues of mice dosed with different MAOs (*n* = 3). Claudin-1 (yellow), occludin (green), ZO-1 (red), and DAPI (blue) (scale: 50 μm) (**a**). Western blot analysis of the expression of (**b**) PPARγ, (**c**) claudin-1, (**d**) occludin, and (**e**) ZO-1 in colon tissues.

In assessing whether PPARγ signaling was associated with improved intestinal barrier function, increased protein expression of tight junction proteins, including claudin-1, occludin, and ZO-1, with MAO administration compared with the DSS group was confirmed by immunofluorescence (Figure 3a). Claudin-1, occludin, and ZO-1 expression was higher with CO treatment than with AO treatment, and they were higher with high-sulfate MAO treatment (ι- and λ-COs) than with κ-COs. Although their expression with high-DP MAO treatment was higher than with low-DP MAO treatment, the differences were not significant.

Western blot analysis showed similar results (Figure 3c–e). Claudin-1, occludin, and ZO-1 expression was significantly upregulated (*p* < 0.01) after MAO administration compared with the DSS group. Their expression with high-sulfate MAO treatment was higher than with κ-CO (*p* < 0.01) and AO treatment (*p* < 0.01), and upregulation was higher with high-DP treatment than with low-DP treatment (except for λ-COs). These results imply the beneficial effects of MAOs on improving intestinal barrier function in mice with colitis.

### 3.8. MAOs Regulated DSS-Induced Gut Microbiota Dysbiosis

**Figure 4:**
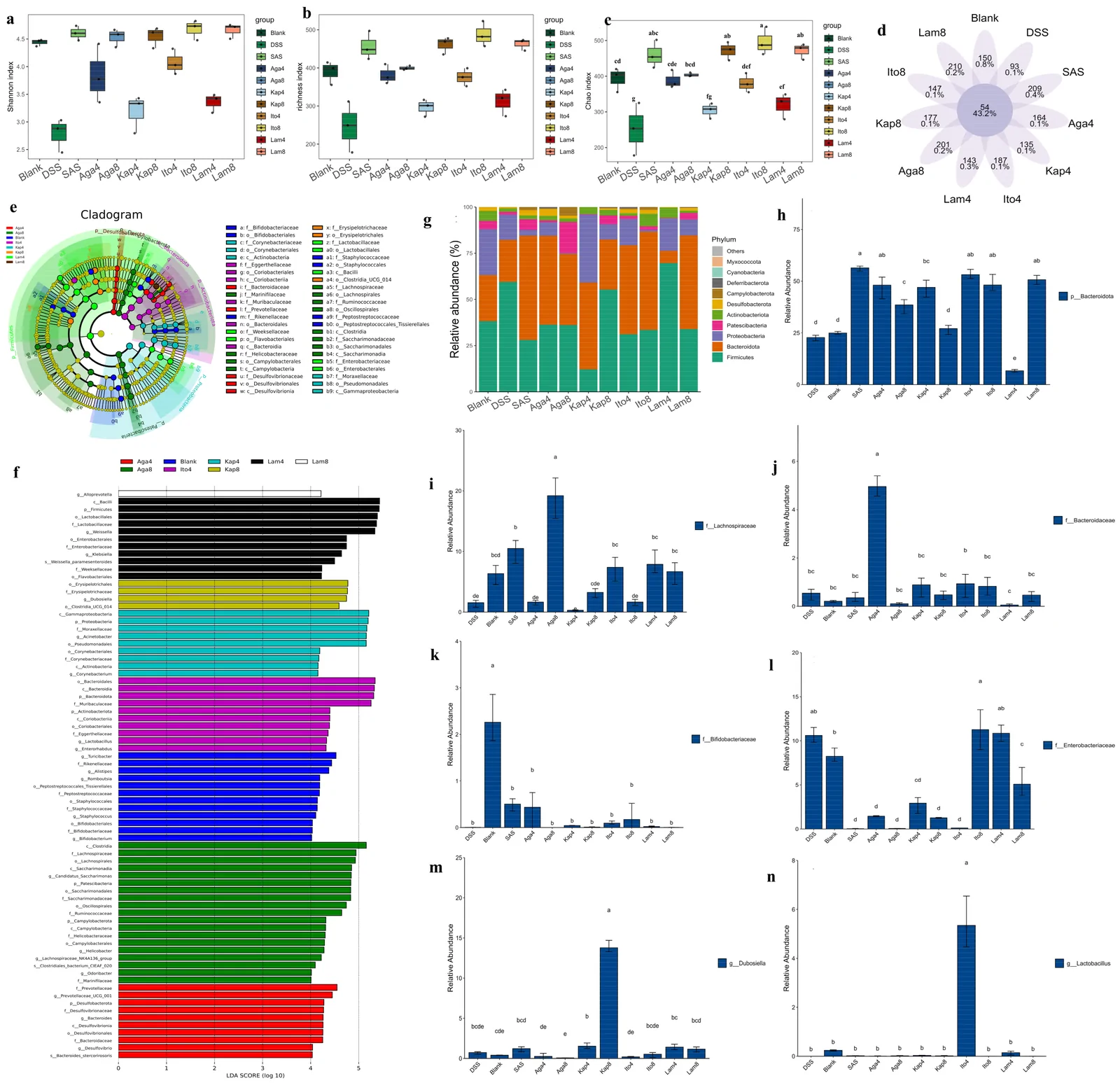
Alpha-diversity index of gut microbiota from mice administered different MAOs (*n* = 3). (**a**) Shannon index; (**b**) richness index; (**c**) Chao1 index; (**d**) venn diagram of different groups; (**e**) a cladogram from LEfSe analysis, showing biomarker taxa of the colonic m icrobes; (**f**) differences in microbial taxa between three groups were analyzed using linear discriminant analysis (LDA) effect sizes, with a LDA score > 3 and a significance of *p* < 0.05 determined by the Wilcoxon signed-rank test; (**g**) the phylum-level composition of gut microbiota from mice administered different MAOs; (**h**) the relative abundance of Bacteroidota in mice administered different MAOs; the relative abundance of gut probiotic bacteria from mice administered different MAOs: (**i**) Lachnospiraceae; (**j**) Bacteroidaceae; (**k**) Bifidobacteriaceae; (**l**) *Dubosiella*; (**m**) *Lactobacillus*; (**n**) the relative abundance of gut potential pathogenic bacteria (Enterobacteriaceae) from mice administered different MAOs. Different lowercase letters in (**h**–**n**) represent significant differences between respective treatments by ANOVA (*p* < 0.05). Note: Aga4, Aga8, Kap4, Kap8, Ito4, Ito8, Lam4, and Lam8 represented A-4, A-8, κ-4, κ-8, ι-4, ι-8, λ-4, and λ-8, respectively.

Compared with the blank control group, gut microbiota alpha-diversity indexes (including Chao1, Shannon, and Richness) in mice with DSS-induced colitis were significantly decreased (*p* < 0.000003, 0.0000008, and 0.0017, respectively, Table S4); all MAO treatments alleviated this decrease (Figure 4a–c). Shannon, richness, and Chao1 indices of CO-treated mice were higher than those of AO-treated mice, while those of high-sulfate CO-treated mice (ι-8 and λ-8) were higher than those of mice treated with κ-8. The alpha-diversity of high-DP MAO-treated groups (A-8, κ-8, ι-8, and λ-8) was higher than that of the low-DP MAO-treated groups (A-4, κ-4, ι-4, and λ-4), as well as that of the blank control group. Although low-DP MAOs also alleviated the decrease in gut microbiota diversity caused by DSS, the alpha-diversity indices of the treated groups were lower than those of the blank control group.

There were 54 shared OTUs among all groups (about 43.2% of all OTUs), with significant differences in the number of unique OTUs between different experimental groups (Figure 4d). The amount of unique OTUs in the DSS-treated group (93) was lower than in all of the MAO-treated groups. There were more unique OTUs in groups treated with higher sulfate and DP MAOs than in those treated with lower sulfate or DP MAOs; λ-8, κ-8, and A-8 groups had 210, 177, and 201, respectively, whereas λ-4, κ-4, and A-4 groups had 143, 135, and 164.

To identify the key taxa contributing to microbial differences, linear discriminant analysis effect size analysis was performed, showing MAO treatment significantly enriched beneficial taxa compared with the DSS model group (Figure 4e,f). The dominant two phyla in the experimental mice were Firmicutes and Bacteroides (Figure 4g). Compared with the blank and DSS groups, MAO treatment groups had significantly increased (*p* < 0.0095) abundance of Bacteroidota (except for ι-4 and κ-8) (Figure 4h), but Firmicutes did not change. Lachnospiraceae abundance was significantly increased (*p* < 0.01) by treatment with A-8, κ-8, ι-4, λ-4, and λ-8 compared with the DSS group (Figure 4i), while A-4 significantly increased (*p* < 0.0042) Bacteroidaceae (Figure 4j). κ-4 significantly increased *Bifidobacterium* abundance, which was decreased by DSS, although its abundance remained lower than that of the blank control group (Figure 4k). *Lactobacillus* was significantly increased by κ-4 and ι-4 compared with the DSS-treated group (*p* < 0.0017) (Figure 4l). A-8, κ-4, ι-4, and κ-8 significantly increased *Dubosiella* compared with the DSS-treated group (*p* < 0.0014) (Figure 4m). The abundance of the potential pathogenic bacteria Enterobacteriaceae in DSS-treated mice was significantly increased compared with the blank control (*p* < 0.0005), but was significantly decreased by MAO treatment (except for ι-4 and ι-8) (Figure 4n).

MAO administration significantly increased gut microbial diversity and probiotic abundance, while decreasing potential pathogens in mice with DSS-induced colitis, with different regulatory effects among different oligosaccharides.

### 3.9. MAOs Regulated Microbially Mediated SCFA Production

**Figure 5:**
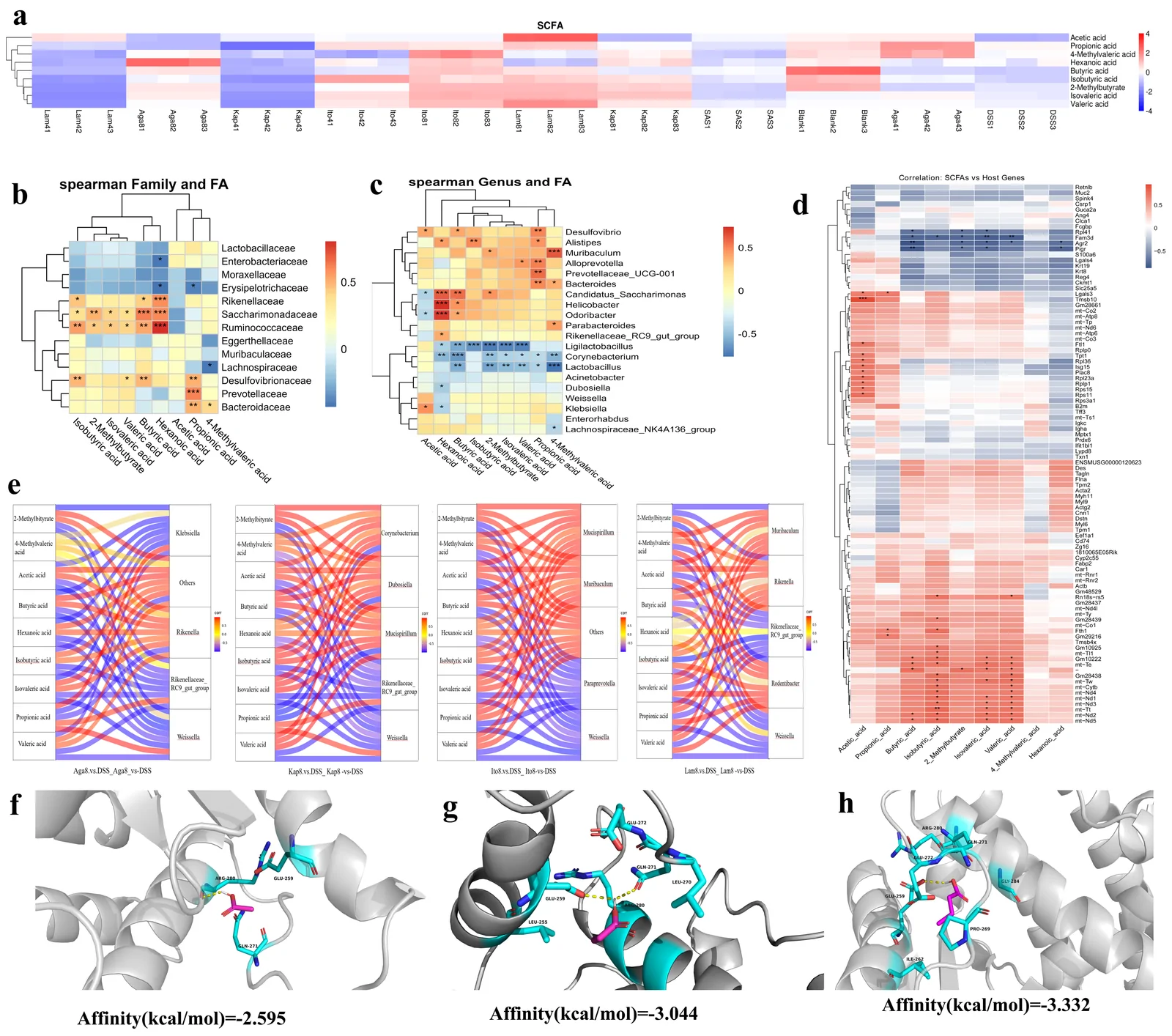
Correlation analysis among gut microbiota, SCFAs and PPARγ signaling pathway (*n* = 3). (**a**) The heatmap of SCFAs distribution in each group; (**b**) Spearman analysis between gut microbiota and SCFAs (family); (**c**) Spearman analysis between gut microbiota and SCFAs (genus); (**d**) Spearman analysis between SCFAs and host genes; * *p* < 0.05, ** *p* < 0.01, *** *p* < 0.01; (**e**) correlation analysis between gut microbiota and SCFAs, A-8 vs. DSS; κ-8 vs. DSS; ι-8 vs. DSS; λ-8 vs. DSS, respectively; modeling of PPARγ binding to SCFAs: (**f**) acetic acid; (**g**) propionic acid; (**h**) butyrate.

The concentrations of seven SCFAs in the colon contents of mice showed that DSS-induced colonic inflammation was associated with a significant reduction in SCFA production (Figure 5a, Table 3). Most MAOs alleviated this effect, with some oligosaccharide treatments resulting in higher SCFA production than that in the blank control group (except for butyric acid). High-sulfate COs (ι- and λ-COs) had greater beneficial effects than κ-COs, and high-DP oligosaccharides had greater beneficial effects than low-DP oligosaccharides. The beneficial effects exerted by high-DP CO treatment (κ-8, ι-8, and λ-8) in mice with colitis were closely associated with increased acetic acid, propionic acid, isobutyric acid, 2-methylbutyrate, and valeric acid production. CO sulfate group content correlated significantly positively with beneficial effects. A-4 significantly improved propionic acid and acetic acid production in comparison with A-8, while A-8 increased butyric acid and 2-methylbutyrate production in comparison with A-4 (Table 3).

The correlation between differential bacterial genera and metabolites was analyzed to clarify the impact of MAOs on the gut microbiota and SCFAs. There was a significant correlation between specific gut probiotics and SCFA abundance. Spearman’s correlation analysis showed that Ruminococcaceae, Sacchrimonadaceae, Rikenellaceae, Desulfovibrionaceae, Prevotellaceae, and Bacteroidaceae were significantly positively correlated with the production of several SCFAs; however, Erysipelotrichaceae, Moraxellaceae, Enterobacteiaceae, and Lactobacillaceae were negatively correlated with SCFA production (Figure 5b,c). Additionally, there is a significant correlation between SCFAs and the host gene expression (Figure 5d).

The interrelationship among MAOs, gut microbiota, and SCFAs was further analyzed (Figure 5e). κ-8 significantly increased *Mucispirillum* and *Dubosiella* abundances, which were positively correlated with almost all SCFAs (excluding propionic acid). ι-8 significantly increased *Mucispirillum* and *Muribaculum* abundances, while λ-8 significantly increased *Rikenella* and *Muribaculum* abundances, which were positively correlated with all SCFAs; A-8 significantly increased *Rikenella* abundance, which was positively correlated with almost all SCFAs. These results also explain why MAOs with higher sulfate content and DP showed the best therapeutic efficacy.

### 3.10. MAO Modulation of PPAR Signaling

**Figure 6:**
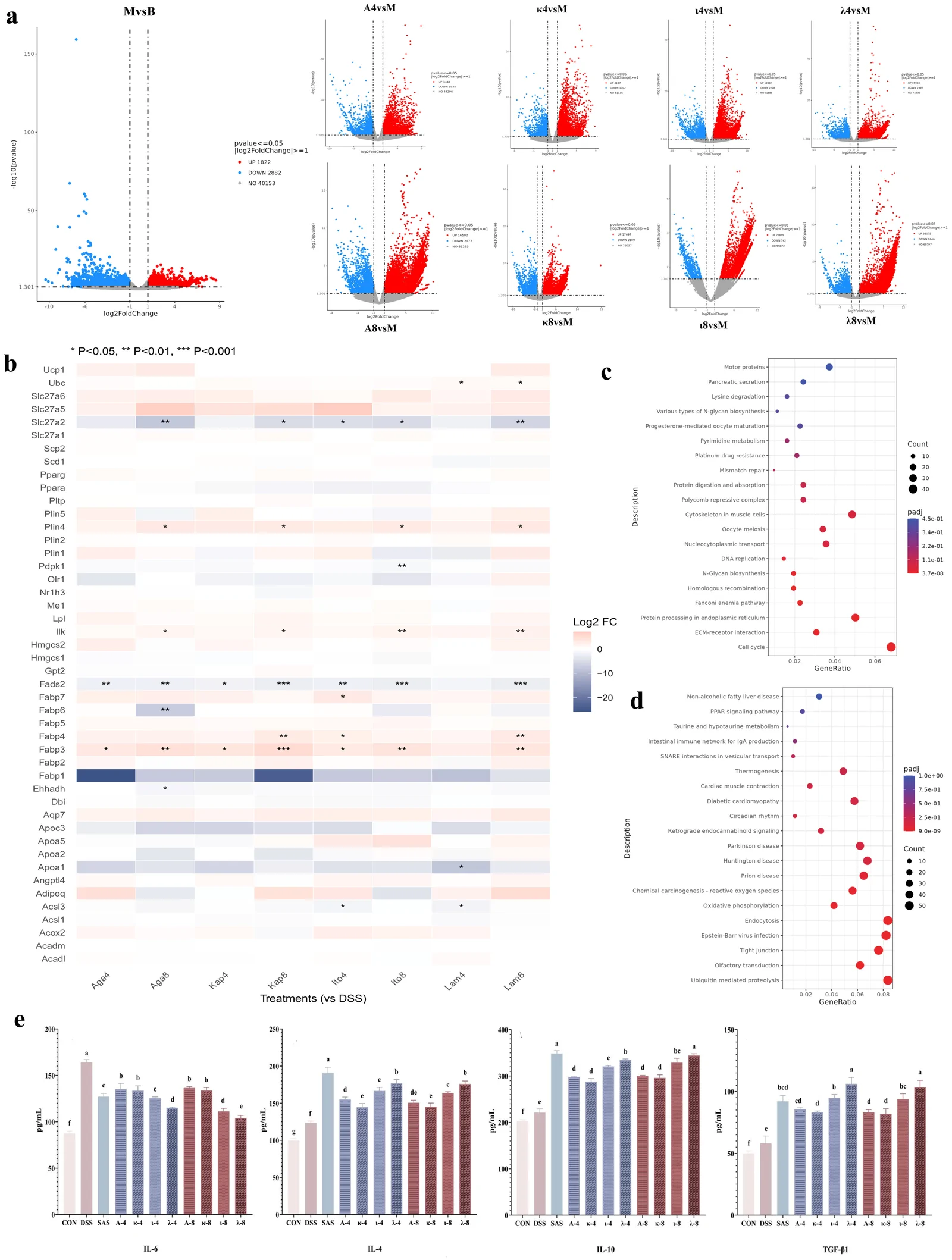
Effect of MAOs on regulating PPARγ signaling in DSS-induced colitis mice (*n* = 4). (**a**) The DEGs of MAO-treated colitis mice compared with DSS-induced colitis mice, (**b**) heatmap of PPARγ downstream signaling genes; KEGG functional enrichment analysis of the DEGs of (**c**) DSS-induced colitis mice and (**d**) MAO-treated colitis mice. (**e**) Inflammatory factor concentrations in colon tissues, including IL-6, IL-4, IL-10, and TGF-β1. Different lowercase letters represent significant differences between respective treatments by ANOVA (*p* < 0.05).

To further delineate the mechanisms underlying the improvement of colitis symptoms in MAO-treated mice, genome-wide transcriptional profiling of colonic tissues was performed with RNA sequencing; a total of 172,107 genes were annotated (Table S5). There were significant transcriptome differences among MAO-treated, control, and DSS-treated mice. Significantly altered genes were identified in the colon of MAO-treated mice compared with DSS-treated mice (Figure 6a). Using the DEGs screening criteria (|log2FC| ≥ 1 and *p* < 0.05), DSS treatment upregulated 1822 genes and downregulated 2882 genes in colon tissues. MAO treatment significantly upregulated gene expression compared to DSS model groups, with the effect of higher DP oligosaccharides being better than that of lower DP oligosaccharides; additionally, the effect of sulfate-containing oligosaccharides was better than that of those without sulfate groups. A4, κ4, ι4, and λ4 upregulated 3448, 4197, 12,832, and 15,903 genes, respectively, while A8, κ8, ι8, and λ8 upregulated 16,502, 17,697, 22,699, and 38,075 genes, respectively (Figure 6a).

Kyoto Encyclopedia of Genes and Genomes pathway analysis of DEGs showed enrichment in a series of signaling pathways (Figure 6c). PPAR signaling and tight junctions were significantly enriched functional pathways related to colitis in MAO-treated mice (Figure 6d). Key genes in the PPARγ signaling pathway were also significantly increased expression in colonic tissues of MAO-treated mice (Figure 6b), including Plin4, Ilk, Fabp3, Fabp4, Fads2, and Slc27a2. Regulation by high-DP oligosaccharides was better than that of lower DP oligosaccharides, and that of high-sulfate oligosaccharides was better than that of those without sulfate groups.

Increased PPARγ protein in colonic tissues of MAO-treated mice was further confirmed by Western blot (Figure 3b). PPARγ expression was significantly upregulated (*p* < 0.01) after MAO administration in comparison with the DSS group. Its expression in colonic tissue of mice treated with high-sulfate MAOs (ι-COs) was higher than in those treated with κ-COs (*p* < 0.01) and AOs (*p* < 0.01), and was higher in high-DP treatment groups than in low-DP treatment groups.

Taken together, MAOs effectively alleviated colitis symptoms by enhancing intestinal barrier function and suppressing inflammatory factors in colonic tissues. The RNA sequencing and Western blot results highlight that MAOs exert these protective effects partly through PPARγ signaling activated by SCFAs.

## 4. Discussion

IBD is a non-specific inflammatory disease of the gut; typical symptoms include abdominal pain, diarrhea, rectal bleeding, and weight loss, though its exact etiology remains unknown. Current clinical treatments mainly use corticosteroids, aminosalicylates, and antibiotics [23]. However, the prolonged use of antibiotics in the management of intestinal conditions is limited because of the risk of severe adverse effects. Because of this, identification of natural anti-inflammatory substances as alternative therapeutic strategies is urgently needed. MAOs are promising natural agents for colitis therapy because of their excellent anti-inflammatory activity and significant potential to modify the gut microbiota. Our results showed that all tested MAOs, particularly those with higher sulfate groups and DP, could significantly reduce the DAI index of mice with colitis compared with the DSS group, indicating an effective therapeutic effect on colitis in mice.

MAOs, including different DP AOs and different types of COs, show excellent anti-inflammatory activity in vitro. Sulfated moieties in saccharides are believed to play an important role in the manifestation of beneficial bioactivity. The anti-inflammatory activity of κ-COs and their desulfurized derivatives were assessed by Yao et al., finding that all the oligosaccharides tested could inhibit the release of tumor necrosis factor-α in microglia activated by LPS [24]; the anti-inflammatory activity of κ-COs was superior to that of desulfurized derivatives, indicating that CO activity is related to sulfate content. Guo et al. also confirmed that the anti-inflammatory activity of COs with more sulfate groups was higher for the same DP [6]. However, the specific relationship between MAO molecular structure and anti-inflammatory activity remained unclear because these experiments were independently carried out at different labs; additionally, data on the anti-inflammatory properties of ι- and λ-COs were lacking.

In the current study, a series of oligosaccharides, including AOs and κ-, ι-, and λ-COs with two different DPs (DP4 and DP8), were prepared by our self-developed MAPD enzymes to explore their anti-inflammatory activity on LPS-induced RAW264.7 macrophages. All oligosaccharides showed excellent anti-inflammatory activities, substantially suppressing the pro-inflammatory cytokine IL-6 in LPS-stimulated RAW264.7 cells and inducing the anti-inflammatory cytokines IL-4, IL-10, and TGF-β1. The anti-inflammatory activity of COs was higher than that of AOs, especially for high-sulfate ι- and λ-COs; CO anti-inflammatory activity was also positively correlated with sulfate group content. Oligosaccharide sulfate group content was closely related and positively correlated to anti-inflammatory activity. Additionally, our examination of the anti-inflammatory activity of ι- and λ-COs with different DPs provides data for their research and application prospects in biomedicine. To date, few studies have reported on the anti-inflammatory activity of ι- and λ-COs. Therefore, our study offers novel insights into the interrelationship of differential sulfate group content with MAO anti-inflammatory activity.

In addition to sulfate group content, hydrolysis and DP are related to MAO biological activity. Guo et al. reported that κ-CO anti-inflammatory activity increased with increasing DP [6]. Wang et al. showed that neoagarotetraose (NA4) exhibited the highest anti-inflammatory activity in RAW264.7 cells, compared with NA2, NA6, and NA8 [7]. The results of our study differ from those of previous reports because there was no significant difference in anti-inflammatory activity between different DPs for the same type of oligosaccharide; this may be because we lacked sufficient oligosaccharide treatment groups with different DPs. Future studies should prepare more oligosaccharides with different DPs to accurately evaluate this relationship.

Typical IBD symptoms present with gut dysbiosis, with a decline in bacterial diversity, relative predominance of potential pathogenic bacteria, and an insufficient amount of protective species [25]. As a consequence, colitis prevention and alleviation by prebiotics are closely related to gut microbiota composition modifications [26]. However, previous research has not comprehensively evaluated the effect of MAO structure on colitis therapeutic efficacy. Our findings indicate that the gut microbiota α-diversity in mice with DSS-induced colitis was significantly decreased, but pretreatment with different MAOs effectively improved it. The effect of COs was higher than that of AOs, that of high-sulfate COs (ι-8 and λ-8) was higher than that of κ-8, and that of high-DP MAOs was higher than that of low-DP MAOs. As potential novel prebiotics, these results indicate that MAOs, especially those with higher sulfate content and high DP, can be used in gut microbiota modulation to influence gut health as a therapy for intestinal inflammation.

Alginate oligosaccharides can act as prebiotics and directly alleviate gut microbial dysbiosis by promoting beneficial microbial growth and decreasing the abundance of those causing or exacerbating inflammation [26,27]. AOs can improve gut microbiota composition by enriching Ruminococcaceae, *Coprococcus*, *Roseburia*, and *Faecalibacterium* abundance [21]. Carrageenan oligosaccharides promote the growth of *Bifidobacterium* and *Lactobacillus* along with greatly enhancing the abundance of mucin-producing Prevotellaceae, and significantly improving SCFA production [28]. The MAO administration significantly increased the abundance of gut probiotics, such as Lachnospiraceae, Bacteroidaceae, *Bifidobacterium*, *Lactobacillus,* and *Dubosiella*; Aga8 significantly increased the abundance of Lachnospiraceae, whereas Kap8 significantly increased the abundance of *Dubosiella*, with Ito4 significantly improving the abundance of *Lactobacillus*. *Lactobacillus* and Bacteroidaceae are commonly used as probiotics and have numerous important functional effects, including immune system modulation, gut mucosal barrier enhancement, pathogenic bacterial inhibition, and balancing gastrointestinal microbiota [29,30]. Lachnospiraceae mainly exists in the intestines of most healthy humans, and is a potentially beneficial bacterium involved in the metabolism of various carbohydrates; they can produce acetic acid and butyric acid through fermentation to provide energy, benefiting host health [21]. *Dubosiella* significantly increased after treatment with MAOs; it regulates host metabolism, enhancing intestinal immunity and improving resistance to inflammatory diseases [31].

The MAO administration decreased potential pathogenic bacteria in DSS-induced colitic mice in comparison with healthy ones. The abundance of the potential pathogenic bacteria Enterobacteriaceae in the gut of DSS-induced mice was significantly increased compared with the blank control, but was significantly inhibited by MAO treatment. Enterobacteriaceae are widely considered to be the most important pathogens in human intestinal infectious diseases, but they are also normal microbiota in the intestines of humans and animals, mainly including *Escherichia*, *Shigella*, and *Salmonella*. AlgO significantly decreases the abundance of *Escherichia*, *Shigella*, and *Peptoniphilus* [21]. Overall, MAOs could modify intestinal microbial composition by stimulating commensal protective bacterial growth and enhancing resistance to disease-inducing bacterial colonization, contributing to colitis improvement. There were different regulatory effects on gut probiotics among different oligosaccharide treatment groups.

MAOs could improve SCFA concentration, which is also an important prebiotic index. Prebiotics are fermented by anaerobic colonic microbiota, producing SCFAs. Acetate and lactate produced by Bifidobacteria and Lactobacilli can be converted to butyrate by other species beneficial to health [32,33]. DSS-induced colonic inflammation was associated with a significant reduction in SCFA production; however, administration of most MAOs alleviated this effect in mice. Higher sulfur content COs (ι- and λ-COs) had increased beneficial effects, as did oligosaccharides with higher DPs. Although our results showed that MAOs with a high degree of polymerization (DP8) exhibited no obvious advantages in in vitro anti-inflammatory assays. However, they exerted significantly better therapeutic effects against colitis in mice compared with low-polymerization-degree MAOs (DP4). We speculate that this phenomenon is mainly attributed to the superior regulatory capacity of DP8 on intestinal microbiota. Specifically, these oligosaccharides significantly elevate the abundance of SCFA-producing probiotics (Figure 5e).

Butyrate is considered the most important gut metabolite in host-microbiome interactions as it exerts potent effects on a variety of colonic mucosal functions, such as inhibiting inflammation and carcinogenesis, exerting antiproliferative properties, inducing apoptosis, reinforcing components of the colonic defense barrier, and decreasing oxidative stress [34]. Butyrate induces PPARγ signaling activation in vitro and in vivo [35]. PPARγ is highly expressed in the colon, and its expression is impaired in patients with ulcerative colitis [36]. DEGs in genome-wide transcriptional profiling of colonic tissues were significantly enriched in PPARγ signaling of MAO-treated mice, and improved PPARγ expression was verified with Western blot. Autodock Vina docking software was used to evaluate interactions between PPARγ ligands with butyrate (Figure 5f–h; Figures S1–S3); although no specific orientation or positional restraints were used to guide butyrate binding, the most favorable solution to the docking was very similar to that of decanoic acid [35]. Based on the transcriptomic and Western blotting (WB) results, we speculate that MAOs exert the protective effects against colitis partly through PPARγ signaling activated by SCFAs.

Given its pivotal role in intestinal homeostasis regulation, PPARγ has been recognized as an important target for IBD treatment [37]. One mechanism by which PPARγ signaling modulates intestinal homeostasis is associated with the modulation of the protective function of colon epithelium cells. By modulating microbiota composition and inducing SCFA production, MAOs can improve intestinal epithelial barrier defense mechanisms. Epithelial barrier function impairment is considered to be one of the initial steps in intestinal inflammation that facilitates antigen bloodstream access from the intestinal lumen, generating an exacerbated immune response [38,39]. Mannuronate oligosaccharide treatment improved the epithelial structure of mice with DSS-induced colitis and significantly increased the expression of tight junction proteins, including claudin-1 and occludin. Odd-numbered AOs can effectively alleviate colitis accompanying type II diabetes mellitus by elevating intestinal junction tightness and integrity [40]. Our study also showed that expression of the tight junction proteins claudin-1, occludin, and ZO-1 was significantly upregulated by MAO administration in comparison with the DSS group. Both immunofluorescence and Western blot showed that high-sulfur MAOs upregulated their expression more than low-sulfur MAOs. These results imply the beneficial effects of MAOs, especially those with high sulfur content, on intestinal tightness and barrier function of mice with colitis.

PPARγ in colonic epithelial cells was shown to protect against experimental IBD [41]. PPARγ ligands suppressed inflammatory gene expression in colonic cell lines by suppressing NF-κB and reducing inflammation in a mouse model of IBD [42,43]. In our results, all MAOs significantly reduced mouse serum IL-6 levels compared with the DSS group while increasing levels of IL-4, IL-10, and TGF-β1. The anti-inflammatory effect of these oligosaccharides was consistent with the results seen in RAW264.7 cells in vitro. The anti-inflammatory activity of COs was higher than that of AOs, and there was a positive correlation between CO anti-inflammatory activity and sulfate group content; however, there was no significant difference in anti-inflammatory activity between oligosaccharides of the same type with different DPs. This anti-inflammatory effect was also confirmed by histological analysis, verifying that MAOs modulate the production of anti-inflammatory cytokines, decreasing intestinal inflammation. It can be hypothesized that these effects are at least partially mediated by PPARγ activated by SCFAs.

## 5. Conclusions

MAOs with higher sulfate content and DP showed improved therapeutic efficacy compared with those with lower contents, we speculate that MAO exerts therapeutic effects by activating PPARγ signaling in IBD progression. However, several important limitations should be acknowledged. First, in animal study, the use of only two DPs (DP4 and DP8) limits the resolution needed to evaluate DP-related effects, especially considering the lack of intermediate or lower/higher molecular weight variants. Second, although our results showed that MAOs regulated DSS-induced gut microbiota dysbiosis by 16S, SCFA production by UHPLC-MS, and PPARγ signaling by genome-wide transcriptional analysis, we still need to evaluate the function of PPARγ antagonists to further validate the molecular mechanism underlying the improvement of colitis symptoms in MAO-treated mice.

The mice colitis model was a classic preclinical tool for evaluating the preventive and therapeutic effects of oligosaccharides on colitis. DSS-induced models can effectively mimic the core pathological injuries of human colitis. Combined with standardized evaluation indicators, these models clearly elucidate the mechanisms by which oligosaccharides exert anti-inflammatory effects, regulate gut microbiota, and repair intestinal barrier function. Furthermore, mice with genetically homogeneous backgrounds and standardized feeding conditions ensure high experimental repeatability, which is suitable for the preliminary efficacy screening and mechanistic exploration of oligosaccharides. Nevertheless, this model presents obvious limitations in clinical treatment. Significant differences in intestinal structure, microbial composition, and immune responses exist between mice and humans. Moreover, the effective dosage for mice far exceeds the tolerable range for humans, which may overestimate the therapeutic efficacy. Future studies should optimize animal models and combine clinical trials to promote the clinical translation of oligosaccharides.

## Figures and Tables

**Table 1:** DAI scoring criteria.

Scores	Body Loss	Stool Consistency	Blood in Stool
0	0	Normal	Normal
1	1–5%	Soft stool	Light blue
2	5–10%	Loose stool	Blue
3	10–15%	Diarrhea	Dark blue
4	>15%		Gross bleeding

**Table 2:** Histological scoring criteria.

Scores	Depth of Invasion	Inflammatory Cells Infiltration	Lymphonodus
0	None	None	None
1	±	Mucosa layer	1
2	+	Submucosa layer	2
3	++	Full	3
4	+++		>3

Note: ±, pericryptal infiltration; +, infiltration into the muscularis mucosae; ++, Widespread infiltration of the muscularis mucosae; +++, submucosal infiltration.

**Table 3:** SCFAs concentration of mice (*n* = 8).

Group	Acetic Acid (μg/g)	Propionic Acid (μg/g)	Butyric Acid (μg/g)	Isobutyric Acid (μg/g)	2-Methylbutyrate (μg/g)	Isovaleric Acid (μg/g)	Valeric Acid (μg/g)	4-Methylvaleric Acid (μg/g)	Hexanoic Acid (μg/g)
Blank	3887.68 ± 34.44 c	674.38 ± 7.89 c	554.41 ± 30.29 a	76.99 ± 3.40 b	37.86 ±0.39 a	41.69 ± 0.17 g	52.00 ± 0.08 d	1.03 ± 0.03 b	2.40 ± 0.03 cd
DSS	2009.69 ± 16.76 h	611.21 ± 0.75 e	319.23 ± 9.67 g	42.41 ± 1.88 f	21.17 ± 0.12 f	38.12 ± 0.18 h	48.44 ± 0.07 e	0.92 ± 0.02 cd	2.19 ± 0.16 e
SAS	3431.09 ± 18.26 de	516.24 ± 1.71 g	525.77 ± 5.48 d	50.09 ± 3.33 e	17.69 ± 0.21 g	31.02 ± 0.16 i	47.61 ± 0.44 e	0.73 ± 0.03 e	2.06 ± 0.09 e
Aga4	3918.84 ± 17.61 c	846.77 ± 4.47 a	366.08 ± 2.91 f	53.48 ± 1.22 e	18.10 ± 0.18 g	45.56 ± 0.24 f	51.88 ± 0.19 d	1.23 ± 0.04 a	2.14 ± 0.10 e
Aga8	2227.17 ± 10.17 g	417.86 ± 1.89 i	459.68 ± 5.18 e	53.37 ± 2.19 e	28.68 ± 0.12 d	48.14 ± 0.13 e	52.15 ± 0.72 d	0.93 ± 0.02 cd	3.37 ± 0.09 a
Kap4	3192.43 ± 19.20 f	235.56 ± 2.55 j	195.69 ± 3.00 i	21.73 ± 0.88 g	10.17 ± 0.02 h	15.03 ± 0.18 j	21.67 ± 0.21 f	0.76 ± 0.05 e	1.68 ± 0.07 f
Kap8	2168.41 ± 31.72 g	587.57 ± 0.96 f	691.05 ± 1.05 c	65.64 ± 1.14 d	32.40 ± 0.40 c	59.25 ± 0.51 c	58.99 ± 0.21 c	0.98 ± 0.03 bc	2.51 ± 0.09 bc
Ito4	3493.37 ± 47.32 d	667.07 ± 1.48 c	438.94 ± 3.27 e	85.2 ± 0.38 a	25.15 ± 0.28 e	52.16 ± 0.40 d	59.40 ± 0.70 c	0.64 ± 0.03 f	2.25 ± 0.17 de
Ito8	3412.78 ± 26.49 e	648.57 ± 2.92 d	813.41 ± 5.37 b	69.41 ± 5.02 cd	36.43 ± 0.17 b	63.98 ± 0.76 a	70.56 ± 0.38 b	1.30 ± 0.05 a	2.62 ± 0.05 b
Lam4	4202.48± 29.51 b	483.54 ± 5.44 h	230.11 ± 1.84 h	24.01 ± 1.08 g	6.95 ± 0.05 i	12.94 ± 0.10 k	20.88 ± 0.13 f	0.63 ± 0.03 f	1.69 ± 0.06 f
Lam8	6845.79 ± 79.10 a	713.35 ± 6.33 b	679.21 ± 10.43 c	72.50 ± 2.15 bc	36.73 ± 0.14 b	63.09 ± 0.39 b	84.44 ± 1.06 a	0.87 ± 0.03 d	1.81 ± 0.06 f

Note: Different lowercase letters represent significant differences between respective treatments by ANOVA (*p* < 0.05).

## Data Availability

The original contributions presented in this study are included in the article/Supplementary Materials. Further inquiries can be directed to the corresponding author.

## Supplementary Materials

The following supporting information can be downloaded at: https://www.mdpi.com/article/10.3390/nu18172750/s1. Table S1: Summary of sequencing data quality for each sample; Table S2: Mapping statistics of samples against the reference genome; Table S3: Data quality control table; Table S4: Alpha diversity index of gut microbiota; Table S5: Annotated genes; Figure S1: Binding energy between PPARγ and acetate ligand; Figure S2: Binding energy between PPARγ and propionate ligand; Figure S3: Binding energy between PPARγ and butyrate ligand.

## Glossary

MAOs, marine algal oligosaccharides; AOs, agaro-oligosaccharides; COs, carrageenan-oligosaccharides; κ-COs, κ-carrageenan-oligosaccharides; ι-COs, ι-carrageenan-oligosaccharides; λ-COs, λ-carrageenan-oligosaccharides; AHG, 3,6-anhydro-L-galactose; DP, degree of polymerization; NDOs, Non-digestible oligosaccharides; MAPD enzymes, macroalgae polysaccharide-degrading enzyme; DSS, Dextran sulfate sodium salt; SAS, Sulfasalazine; SCFAs, Short Chain Fatty Acids.
