# Supplementary material for: Gut Microbiota-Derived SCFAs Modulated by Different Structures of Marine Algal Oligosaccharides Alleviate Colitis Through PPARγ Signaling Activation
Source: Nutrients. 2026 Aug 22;18(17):2750. doi: 10.3390/nu18172750 (PMC13567188; doi:10.3390/nu18172750)
Supplement: Supplementary file 1 [file nutrients-18-02750-s001.zip › nutrients-4405591-supplementary/nutrients-4405591-supplementary.pdf]

Supplementary Information

Table S1 Summary of sequencing data quality for each sample

Table S2 Mapping statistics of samples against the reference genome

Table S3 Data quality control table

Table S4 Alpha diversity index of gut microbiota

Fig.S1 Binding energy between PPARγ and acetate ligand

Fig.S2 Binding energy between PPARγ and propionate ligand

Fig.S3 Binding energy between PPARγ and butyrate ligand

Table S1 Summary of sequencing data quality for each sample

**Sample**	**Library**	**Raw reads**	**Raw bases**	**Clean reads**	**Clean bases**	**Error rate**	**Q20**	**Q30**	**GC pct**
Blank.1	FRRB250410837-1r	44404380	6.66G	42812916	6.42G	0.01	99.58	98.12	41.45
Blank.2	FRRB250410838-1r	47569050	7.14G	46007454	6.9G	0.01	99.58	98.17	40.37
Blank.3	FRRB25H004185-1a	47695074	7.15G	44623732	6.69G	0.01	99.28	96.68	43.65
DSS.1	FRRB250410839-1r	47853646	7.18G	46496854	6.97G	0.01	99.49	97.8	48.14
DSS.2	FRRB250410840-1r	43335290	6.5G	40264646	6.04G	0.01	99.31	97.23	49.59
DSS.3	FRRB250410841-1r	44094236	6.61G	41950188	6.29G	0.01	99.32	97.27	48.88
DSS.4	FRRB250410842-1r	43614568	6.54G	41366742	6.21G	0.01	99.34	97.36	48.79
Aga4.1	FRRB250410847-1r	49910710	7.49G	45794632	6.87G	0.01	99.54	98.07	42.25
Aga4.2	FRRB250410848-1r	49290170	7.39G	45988776	6.9G	0.01	99.13	96.9	46.17

Aga4.3	FRRB250410849-1r	49765664	7.46G	46719998	7.01G	0.01	99.43	97.74	45.06
Aga4.4	FRRB250410850-1r	43298808	6.49G	42549380	6.38G	0.01	99.62	98.22	40.14
Kap4.1	FRRB250410851-1r	47128170	7.07G	45468980	6.82G	0.01	99.58	98.13	41.14
Kap4.2	FRRB250410852-1r	49051884	7.36G	48030772	7.2G	0.01	99.53	97.97	42.12
Kap4.3	FRRB250410853-1r	48304732	7.25G	46629408	6.99G	0.01	99.39	97.67	46.38
Kap4.4	FRRB250410854-1r	53766344	8.06G	52874368	7.93G	0.01	99.48	97.81	45.26
Ito4.1	FRRB250410855-1r	46959802	7.04G	46125696	6.92G	0.01	99.61	98.24	41.02
Ito4.2	FRRB250410856-1r	54812628	8.22G	53659718	8.05G	0.01	99.46	97.82	43.35
Ito4.3	FRRB250410857-1r	47706000	7.16G	47524584	7.13G	0.01	99.36	97.66	43.55
Ito4.4	FRRB250410858-1r	45831590	6.87G	44976014	6.75G	0.01	99.55	97.99	43.31
Lam4.1	FRRB250410859-1r	47248844	7.09G	45814786	6.87G	0.01	99.51	97.96	44.15
Lam4.2	FRRB250410860-1r	46917354	7.04G	46792764	7.02G	0.01	99.38	97.67	43.69
Lam4.3	FRRB250410861-1r	48327262	7.25G	47132118	7.07G	0.01	99.53	98.06	41.59
Lam4.4	FRRB250410862-1r	47884078	7.18G	46785772	7.02G	0.01	99.47	97.87	42.19
Aga8.1	FRRB250410863-1r	49014816	7.35G	48002052	7.2G	0.01	99.52	97.94	43.32
Aga8.2	FRRB250410864-1r	50049072	7.51G	49141390	7.37G	0.01	99.35	97.61	40.74
Aga8.3	FRRB250410865-1r	47179360	7.08G	46514938	6.98G	0.01	99.48	97.9	45.83
Kap8.1	FRRB250410866-1r	38706430	5.81G	37423214	5.61G	0.01	99	96.74	40.2
Kap8.2	FRRB250410867-1r	46868594	7.03G	44947788	6.74G	0.01	98.55	96.14	41.26
Kap8.3	FRRB250410868-1r	46147340	6.92G	44153090	6.62G	0.01	99.03	97.09	41.43

Ito8.1	FRRB250410869-1r	47482294	7.12G	46554086	6.98G	0.01	99.38	97.57	43.81
Ito8.2	FRRB250410870-1r	50489504	7.57G	49403142	7.41G	0.01	99.33	97.51	45.38
Ito8.3	FRRB250410871-1r	50767354	7.62G	49873124	7.48G	0.01	99.51	97.9	41.66
Ito8.4	FRRB250410872-1r	47510438	7.13G	46724896	7.01G	0.01	99.6	98.25	42.09
Lam8.1	FRRB250410873-1r	44965720	6.74G	44134912	6.62G	0.01	99.37	97.61	40.47
Lam8.2	FRRB250410874-1r	43481204	6.52G	42623906	6.39G	0.01	99.35	97.31	41.99
Lam8.3	FRRB250410875-1r	46687352	7G	44660040	6.7G	0.01	99.2	97.24	43.66
Lam8.4	FRRB250410876-1r	43670648	6.55G	42860572	6.43G	0.01	99.56	98.05	40.8

Table S2 Mapping statistics of samples against the reference genome

**sample**	**total reads**	**Total map**	**Unique map**	**Multi map**	**read1 map**	**read2 map**	**Positive map**	**Negative map**	**Splice map**	**Unsplice map**	**Proper map**
Blank.1	42812916	40397176(94.36%)	25832941(60.34%)	14564235(34.02%)	12937310(30.22%)	12895631(30.12%)	12905959(30.15%)	12926982(30.19%)	3778905(8.83%)	22054036(51.51%)	25598562(59.79%)
Blank.2	46007454	45404569(98.69%)	28630278(62.23%)	16774291(36.46%)	14334733(31.16%)	14295545(31.07%)	14303981(31.09%)	14326297(31.14%)	3073888(6.68%)	25556390(55.55%)	28356740(61.64%)
Blank.3	44623732	41372870(92.71%)	32233443(72.23%)	9139427(20.48%)	16163862(36.22%)	16069581(36.01%)	16101487(36.08%)	16131956(36.15%)	7414287(16.62%)	24819156(55.62%)	31789248(71.24%)
DSS.1	46496854	45765917(98.43%)	42378486(91.14%)	3387431(7.29%)	21217788(45.63%)	21160698(45.51%)	21177665(45.55%)	21200821(45.6%)	15757208(33.89%)	26621278(57.25%)	41878898(90.07%)

DSS.2	40264646	39564737(98.26%)	36426087(90.47%)	3138650(7.8%)	18270269(45.38%)	18155818(45.09%)	18193985(45.19%)	18232102(45.28%)	14972667(37.19%)	21453420(53.28%)	36016016(89.45%)
DSS.3	41950188	41341648(98.55%)	38526688(91.84%)	2814960(6.71%)	19310835(46.03%)	19215853(45.81%)	19250038(45.89%)	19276650(45.95%)	14472646(34.5%)	24054042(57.34%)	38093920(90.81%)
DSS.4	41366742	40743102(98.49%)	38132616(92.18%)	2610486(6.31%)	19109986(46.2%)	19022630(45.99%)	19053380(46.06%)	19079236(46.12%)	14378975(34.76%)	23753641(57.42%)	37699648(91.14%)
Aga4.1	45794632	44847939(97.93%)	31584683(68.97%)	13263256(28.96%)	15814700(34.53%)	15769983(34.44%)	15783189(34.47%)	15801494(34.51%)	5321445(11.62%)	26263238(57.35%)	31228330(68.19%)
Aga4.2	45988776	44934804(97.71%)	39689435(86.3%)	5245369(11.41%)	19891597(43.25%)	19797838(43.05%)	19827793(43.11%)	19861642(43.19%)	10238447(22.26%)	29450988(64.04%)	38981238(84.76%)
Aga4.3	46719998	45378253(97.13%)	36159017(77.4%)	9219236(19.73%)	18106906(38.76%)	18052111(38.64%)	18072557(38.68%)	18086460(38.71%)	9498324(20.33%)	26660693(57.06%)	35604754(76.21%)
Aga4.1	42549380	41703911(98.01%)	25527676(60.0%)	16176235(38.02%)	12783777(30.04%)	12743899(29.95%)	12751261(29.97%)	12776415(30.03%)	2686202(6.31%)	22841474(53.68%)	25314896(59.5%)
Kap4.1	45468980	44900210(98.75%)	29509436(64.9%)	15390774(33.85%)	14770291(32.48%)	14739145(32.42%)	14748193(32.44%)	14761243(32.46%)	3968636(8.73%)	25540800(56.17%)	29237666(64.3%)
Kap4.2	48030772	47086040(98.03%)	32246133(67.14%)	14839907(30.9%)	16142326(33.61%)	16103807(33.53%)	16110164(33.54%)	16135969(33.6%)	4709865(9.81%)	27536268(57.33%)	31764714(66.13%)
Kap4.3	46629408	45752241(98.12%)	40061926(85.92%)	5690315(12.2%)	20063700(43.03%)	19998226(42.89%)	20026228(42.95%)	20035698(42.97%)	11530972(24.73%)	28530954(61.19%)	39508672(84.73%)
Kap4.4	52874368	51950268(98.25%)	42705381(80.77%)	9244887(17.48%)	21381219(40.44%)	21324162(40.33%)	21346705(40.37%)	21358676(40.4%)	10985772(20.78%)	31719609(59.99%)	42144046(79.71%)

Ito4.1	46125696	45385669(98.4%)	29295124(63.51%)	16090545(34.88%)	14667276(31.8%)	14627848(31.71%)	14630466(31.72%)	14664658(31.79%)	3705406(8.03%)	25589718(55.48%)	28931368(62.72%)
Ito4.2	53659718	52038096(96.98%)	40066893(74.67%)	11971203(22.31%)	20060214(37.38%)	20006679(37.28%)	20018977(37.31%)	20047916(37.36%)	7425016(13.84%)	32641877(60.83%)	39441380(73.5%)
Ito4.3	47524584	46304320(97.43%)	34829601(73.29%)	11474719(24.14%)	17448044(36.71%)	17381557(36.57%)	17397955(36.61%)	17431646(36.68%)	7170897(15.09%)	27658704(58.2%)	34378290(72.34%)
Ito4.4	44976014	41805489(92.95%)	27281941(60.66%)	14523548(32.29%)	13662561(30.38%)	13619380(30.28%)	13568922(30.17%)	13713019(30.49%)	3227780(7.18%)	24054161(53.48%)	26205482(58.27%)
Lam4.1	45814786	44992102(98.2%)	34030519(74.28%)	10961583(23.93%)	17043382(37.2%)	16987137(37.08%)	17003787(37.11%)	17026732(37.16%)	8960886(19.56%)	25069633(54.72%)	33624218(73.39%)
Lam4.2	46792764	44750758(95.64%)	32697627(69.88%)	12053131(25.76%)	16374249(34.99%)	16323378(34.88%)	16333293(34.91%)	16364334(34.97%)	8410174(17.97%)	24287453(51.9%)	32290968(69.01%)
Lam4.3	47132118	34434419(73.06%)	24672632(52.35%)	9761787(20.71%)	12359535(26.22%)	12313097(26.12%)	12324023(26.15%)	12348609(26.2%)	4325708(9.18%)	20346924(43.17%)	24390792(51.75%)
Lam4.4	46785772	45385927(97.01%)	31143768(66.57%)	14242159(30.44%)	15598232(33.34%)	15545536(33.23%)	15547335(33.23%)	15596433(33.34%)	4678750(10.0%)	26465018(56.57%)	30522420(65.24%)
Aga8.1	48002052	47182605(98.29%)	34478187(71.83%)	12704418(26.47%)	17258355(35.95%)	17219832(35.87%)	17225832(35.89%)	17252355(35.94%)	6122481(12.75%)	28355706(59.07%)	34033696(70.9%)
Aga8.2	49141390	47188395(96.03%)	31089973(63.27%)	16098422(32.76%)	15544266(31.63%)	15545707(31.63%)	15523343(31.59%)	15566630(31.68%)	3739145(7.61%)	27350828(55.66%)	30366234(61.79%)
Aga8.3	46514938	45747512(98.35%)	38265500(82.26%)	7482012(16.09%)	19161938(41.2%)	19103562(41.07%)	19120999(41.11%)	19144501(41.16%)	9129154(19.63%)	29136346(62.64%)	37758260(81.17%)

Kap 8.1	37423214	34701916(92.73%)	25335050(67.7%)	9366866(25.03%)	12724215(34.0%)	12610835(33.7%)	12647508(33.8%)	12687542(33.9%)	2436749(6.51%)	22898301(61.19%)	24550526(65.6%)
Kap 8.2	44947788	42494444(94.54%)	28440567(63.27%)	14053877(31.27%)	14357321(31.94%)	14083246(31.33%)	14040223(31.24%)	14400344(32.04%)	4045477(9.0%)	24395090(54.27%)	27498176(61.18%)
Kap 8.3	44153090	42547387(96.36%)	28839959(65.32%)	13707428(31.05%)	14486499(32.81%)	14353460(32.51%)	14333975(32.46%)	14505984(32.85%)	3963882(8.98%)	24876077(56.34%)	28307852(64.11%)
Ito8.1	46554086	43921588(94.35%)	33214735(71.35%)	10706853(23.0%)	16640381(35.74%)	16574354(35.6%)	16558455(35.57%)	16656280(35.78%)	5761206(12.38%)	27453529(58.97%)	32180244(69.12%)
Ito8.2	49403142	46561955(94.25%)	40006051(80.98%)	6555904(13.27%)	20029904(40.54%)	19976147(40.43%)	19984060(40.45%)	20021991(40.53%)	8576240(17.36%)	31429811(63.62%)	39218980(79.39%)
Ito8.3	49873124	46906310(94.05%)	32450406(65.07%)	14455904(28.99%)	16246152(32.57%)	16204254(32.49%)	16213710(32.51%)	16236696(32.56%)	4343788(8.71%)	28106618(56.36%)	31940820(64.04%)
Ito8.4	46724896	46011921(98.47%)	31649354(67.74%)	14362567(30.74%)	15843234(33.91%)	15806120(33.83%)	15814204(33.85%)	15835150(33.89%)	4980417(10.66%)	26668937(57.08%)	31307118(67.0%)
Lam 8.1	44134912	42951634(97.32%)	28022023(63.49%)	14929611(33.83%)	14042714(31.82%)	13979309(31.67%)	13983947(31.68%)	14038076(31.81%)	3320122(7.52%)	24701901(55.97%)	27431136(62.15%)
Lam 8.2	42623906	41203836(96.67%)	28661101(67.24%)	12542735(29.43%)	14367198(33.71%)	14293903(33.53%)	14305522(33.56%)	14355579(33.68%)	4893818(11.48%)	23767283(55.76%)	28094102(65.91%)
Lam 8.3	44660040	41214864(92.29%)	31462038(70.45%)	9752826(21.84%)	15749989(35.27%)	15712049(35.18%)	15709301(35.18%)	15752737(35.27%)	5346089(11.97%)	26115949(58.48%)	30547908(68.4%)
Lam 8.4	42860572	42168854(98.39%)	27270689(63.63%)	14898165(34.76%)	13654985(31.86%)	13615704(31.77%)	13617535(31.77%)	13653154(31.85%)	2820151(6.58%)	24450538(57.05%)	26898556(62.76%)

Table S3 Data quality control table

Sample	RawPE	Combined	Qualified	Nochime	Base(nt)	Avglen(nt)	GC	Q20	Q30
Blank.1	97369	96559	93721	54198	22863798	421.86	52.99%	98.16%	93.96%
Blank.2	100272	99470	96762	76820	32423933	422.08	52.90%	98.42%	94.65%
Blank.3	89355	88590	85843	72859	30735902	421.85	52.94%	98.26%	94.29%
DSS.1	60984	60557	58737	44429	18946380	426.44	51.06%	98.17%	94.05%
DSS.2	87120	86393	83582	73616	31376782	426.22	51.35%	98.30%	94.31%
DSS.3	110064	109240	106040	92736	39498386	425.92	51.39%	98.39%	94.60%
SAS.1	103438	102630	100007	49153	20607069	419.24	54.47%	98.12%	93.98%
SAS.2	105971	105026	101930	67118	28085357	418.45	54.68%	98.16%	94.13%
SAS.3	106365	105606	102843	91786	38418840	418.57	54.05%	98.39%	94.71%
Aga4.1	102178	101408	98792	55865	23711814	424.45	52.83%	98.03%	93.61%
Aga4.2	105474	104487	101367	73374	31087783	423.69	53.08%	98.26%	94.29%
Aga4.3	106451	105409	102686	85883	36325632	422.97	53.66%	98.28%	94.35%
Kap4.1	103731	102859	100647	60492	25760651	425.85	53.95%	98.01%	93.52%
Kap4.2	102698	101551	98953	84538	35972384	425.52	54.10%	98.34%	94.44%
Kap4.3	104863	103917	101225	85317	36290338	425.36	54.23%	98.33%	94.51%
Ito4.1	116623	115683	112633	54864	22930976	417.96	54.98%	97.98%	93.66%
Ito4.2	116338	115085	111799	93048	38871751	417.76	54.28%	97.93%	93.51%
Ito4.3	102432	101537	98967	89708	37452553	417.49	53.76%	98.40%	94.65%
Lam4.1	106570	105655	102646	72607	30907598	425.68	50.98%	98.21%	94.08%
Lam4.2	105794	105027	101696	92465	39258933	424.58	51.10%	98.27%	94.22%
Lam4.3	102536	101456	97900	88404	37593304	425.24	51.05%	98.10%	93.70%
Aga8.1	114727	113866	111322	61894	25473017	411.56	52.66%	98.31%	94.34%

Aga8.2	103089	102143	99394	77762	32096503	412.75	53.71%	98.39%	94.64%
Aga8.3	103680	102679	100158	82792	34199276	413.07	53.76%	98.47%	94.85%
Kap8.1	106531	105671	102529	57678	24250847	420.45	52.63%	98.03%	93.67%
Kap8.2	103630	102680	99622	85588	35932747	419.83	52.75%	98.28%	94.33%
Kap8.3	87932	87140	84580	71580	30054335	419.87	52.66%	98.34%	94.42%
Ito8.1	110719	109775	106875	91915	38908479	423.31	53.19%	98.35%	94.56%
Ito8.2	102921	102169	99521	52686	22345667	424.13	53.52%	98.04%	93.75%
Ito8.3	85980	84958	82423	59268	25111736	423.7	53.00%	98.24%	94.10%
Lam8.1	103876	102868	99969	51480	21732298	422.15	53.31%	98.10%	93.88%
Lam8.2	119076	117870	114246	100353	42258232	421.1	53.48%	98.26%	94.30%
Lam8.3	117570	116217	112985	95065	40053119	421.32	53.55%	98.32%	94.37%

Table S4

Metric	P	Blank	DSS	SAS	Aga4	Aga8	Kap4	Kap8	Ito4	Ito8	Lam4	Lam8
Chao1	2.835 e-06	403.65 ± 38.85 abc	254.85 ± 71.91 d	466.5 ± 32.28 ab	401.39 ± 28.29 abc	416.22 ± 8.17 abc	313.92 ± 33.32 cd	476.77 ± 24.62 ab	383.14 ± 27.49 bc	498.26 ± 39.83 a	329.27 ± 34.55 cd	476.5 ± 17.33 ab
richniess	1.174 e-03	393 ± 32.74 ab	248.33 ± 66.83 a	457.67 ± 35.84 b	388 ± 28.16 ab	403.67 ± 4.51 ab	301.33 ± 26.1 a	465.33 ± 21.94 b	377 ± 23.52 ab	490.67 ± 36.83 b	316 ± 37 a	466.33 ± 18.5 b
shannon	8.415 e-07	6.4 ± 0.09 ab	4.02 ± 0.44 e	6.65 ± 0.19 a	5.56 ± 0.78 bcd	6.53 ± 0.23 ab	4.6 ± 0.5 de	6.57 ± 0.28 ab	5.88 ± 0.33 abc	6.75 ± 0.26 a	4.84 ± 0.25 cde	6.73 ± 0.2 a

```
Computing Vina grid ... done.
Performing docking (random seed: 1735277651) ...
0%   10   20   30   40   50   60   70   80   90   100%
|----|----|----|----|----|----|----|----|----|----|
***************************************************

mode |   affinity | dist from best mode
     | (kcal/mol) | rmsd l.b.| rmsd u.b.
-----+------------+----------+----------
   1       -2.595          0          0
   2       -2.594      1.949      2.341
   3       -2.548      1.299      1.707
   4       -2.337      8.458      8.861
   5       -2.112      8.661      9.033
   6       -2.106      2.456       2.67
   7       -2.079      8.492      8.801
   8       -2.021      8.517       8.78
   9       -2.015      8.482      8.684
  10       -1.978       10.9      11.18
  11       -1.849      9.148      9.365
  12       -1.685      9.082      9.295
  13       -1.635      11.41      11.64
  14         -1.5      10.72      11.21
  15       -1.434      9.447      9.681
  16       -1.393      10.38       10.8
  17       -1.325      11.05      11.44
  18       -1.297      9.091      9.245
  19       -1.292      10.32      10.77
  20        -1.19      9.167      9.207

D:\docking>
```

Fig.S1 Binding energy between PPARγ and acetate ligand

```
Computing Vina grid ...  done.
Performing docking (random seed: -1415082245) ...
0%   10   20   30   40   50   60   70   80   90   100%
|----|----|----|----|----|----|----|----|----|----|
***************************************************

mode |   affinity | dist from best mode
     | (kcal/mol) | rmsd l.b.| rmsd u.b.
-----+------------+----------+----------
   1       -3.044          0          0
   2       -2.998     0.9787      1.147
   3       -2.957       1.88      2.817
   4       -2.813      2.173      2.597
   5       -2.509      4.244      4.434
   6       -2.507      9.811      10.69
   7       -2.429      2.843      3.628
   8       -2.428      1.547      1.803
   9       -2.409      2.187      2.719
  10       -2.291      9.717      10.49
  11       -2.267      9.847      10.71
  12       -2.231      1.769      2.637
  13       -2.124      2.533      2.693
  14        -2.09      2.549      3.341
  15       -2.062      10.19      10.87
  16       -2.051      9.785      10.38
  17       -1.914       11.6      12.11
  18       -1.801      9.909      10.46
  19       -1.728      12.13       12.6
  20       -1.697      10.37      11.11

D:\docking>
```

Fig.S2 Binding energy between PPARγ and propionate ligand

mode	affinity (kcal/mol)	dist from best mode rmsd l. b.	rmsd u. b.
1	-3. 332	0	0
2	-3. 321	2. 286	3. 663
3	-3. 296	2. 114	2. 422
4	-3. 145	2. 035	3. 149
5	-2. 999	2. 551	3. 252
6	-2. 596	2. 395	3. 283
7	-2. 583	8. 749	9. 086
8	-2. 581	8. 572	8. 814
9	-2. 503	8. 605	8. 811
10	-2. 421	8. 549	8. 882
11	-2. 068	8. 851	9. 353
12	-1. 983	9. 291	9. 754
13	-1. 954	8. 68	9. 007
14	-1. 926	9. 049	9. 7
15	-1. 893	9. 483	10. 25
16	-1. 887	9. 114	9. 509
17	-1. 831	11. 47	12. 36
18	-1. 698	10. 56	11. 1
19	-1. 624	10. 05	10. 47
20	-0. 1266	2. 06	2. 54

Fig.S3 Binding energy between PPARγ and butyrate ligand
